# Introducing Brønsted acid sites to accelerate the bridging-oxygen-assisted deprotonation in acidic water oxidation

**DOI:** 10.1038/s41467-022-32581-w

**Published:** 2022-08-18

**Authors:** Yunzhou Wen, Cheng Liu, Rui Huang, Hui Zhang, Xiaobao Li, F. Pelayo García de Arquer, Zhi Liu, Youyong Li, Bo Zhang

**Affiliations:** 1 State Key Laboratory of Molecular Engineering of Polymers, Department of Macromolecular Science, Fudan University, Shanghai, 200438 China; 2 Institute of Functional Nano & Soft Materials (FUNSOM) and Jiangsu Key Laboratory for Carbon-Based Functional Materials & Devices, Soochow University, Suzhou, 215123 China; 3 State Key Laboratory of Functional Materials for Informatics, Shanghai Institute of Microsystem and Information Technology, Chinese Academy of Sciences, Shanghai, 200050 China; 4 ICFO - Institut de Ciències Fotòniques, The Barcelona Institute of Science and Technology, Barcelona, 08860 Spain; 5 School of Physical Science and Technology and Center for Transformative Science, ShanghaiTech University, Shanghai, 201210 China

**Keywords:** Renewable energy, Nanoscale materials, Electrocatalysis

## Abstract

Oxygen evolution reaction (OER) consists of four sequential proton-coupled electron transfer steps, which suffer from sluggish kinetics even on state-of-the-art ruthenium dioxide (RuO_2_) catalysts. Understanding and controlling the proton transfer process could be an effective strategy to improve OER performances. Herein, we present a strategy to accelerate the deprotonation of OER intermediates by introducing strong Brønsted acid sites (e.g. tungsten oxides, WO_x_) into the RuO_2_. The Ru-W binary oxide is reported as a stable and active iridium-free acidic OER catalyst that exhibits a low overpotential (235 mV at 10 mA cm^−2^) and low degradation rate (0.014 mV h^−1^) over a 550-hour stability test. Electrochemical studies, in-situ near-ambient pressure X-ray photoelectron spectroscopy and density functional theory show that the W-O-Ru Brønsted acid sites are instrumental to facilitate proton transfer from the oxo-intermediate to the neighboring bridging oxygen sites, thus accelerating bridging-oxygen-assisted deprotonation OER steps in acidic electrolytes. The universality of the strategy is demonstrated for other Ru-M binary metal oxides (M = Cr, Mo, Nb, Ta, and Ti).

## Abstract (web summary)

While water electrolysis devices represent a technology for renewable energy, there are few stable catalysts that survive the acidic conditions. Here, authors enhance acidic oxygen evolution by introducing strong Brønsted acid sites into RuO_2_ to accelerate bridging-oxygen-assisted deprotonation.

## Introduction

The oxygen evolution reaction (OER) is one of the pivotal reactions in electrochemical energy storage and conversion^1^, which is the anodic reaction in water electrolysis^2^, CO_2_ electroreduction^3^, metal-air batteries^4,5^, electro-winning^6^, etc. Specifically, the proton-exchange membrane (PEM) water electrolysis devices require OER catalysts with high activity and corrosion resistance in acidic environments^7^. However, the sluggish kinetics of OER leads to high overpotentials. Even for well-studied benchmark ruthenium oxide (RuO_2_) catalysts^8^, the long-term catalytic activity is far less than the targets required for large-scale renewable energy conversion devices^7^.

The conventional OER mechanism on RuO_2_ can be described as four sequential proton-coupled electron transfer (PCET) deprotonation steps, in which the protons are desorbed from the oxo-intermediates (and water molecular) and released into the electrolyte directly^9^. In alkaline solutions, the abundant OH^−^ ions assist this direct deprotonation process^10,11^. In acidic conditions, however, direct deprotonation becomes difficult due to the high proton concentration in the electrolyte. Accelerating the deprotonation of oxo-intermediates is one promising direction to improve OER kinetics in acidic electrolytes.

Recent research on RuO_2_ and IrO_2_ systems showed that the bridging oxygen (denoted as O_bri_ in the following text, a schematic of different oxygen sites is shown in Supplementary Fig. 1) can accept protons from H_2_O or OER intermediates, providing a new possible path to OER intermediates deprotonation through the participation of O_bri_^8,12,13^. A recent study on single-crystal RuO_2_ has shown that on the RuO_2_ (110) facet, the OOH* intermediate transfers one proton to the adjacent O_bri_, forming protonated bridging oxygen (OH_bri_) and the deprotonation of OH_bri_ is the rate-determining step (RDS)^8,13^. By switching the facet orientation, the proton adsorption energetics on O_bri_ can be tuned, thus altering the OER activity. However, the facet engineering approach is intrinsically limited to single crystals. Implementing this fundamental finding to improve the performance of industrially scalable and stable catalysts is still an open challenge^14^. Strategies to regulate the proton adsorption/desorption energetics on O_bri_ and further accelerate this bridging-oxygen-assisted deprotonation (BOAD) process are urgently needed for the development of acidic OER electrocatalysts.

The deprotonation of surface OH_bri_ sites can be described by the Brønsted-type acidity. In heterogeneous solid-acid catalysts such as zeolites^15^, supported catalysts^16^, and metal-organic frameworks^17^, the acidity and density of Brønsted acid sites strongly affect the activity and mechanism of dehydration, isomerization, and cracking reactions. Similarly, it is rational that the deprotonation energetics of surface O_bri_ sites can be optimized by precisely tuning the Brønsted acidity of OH_bri_, thus the OER kinetics.

We, therefore, hypothesized that a tailored introduction of strong Brønsted acid sites into the RuO_2_ lattice could optimize the deprotonation energetics of O_bri_ on the catalytic surface. We implemented this strategy through the selective incorporation of tungsten (W) oxides –which have versatile crystal structure^18^, acid stability^19^, and unique proton adsorption^20,21^–to produce flexible surface O_bri_ sites on RuO_2_.

In this work, we successfully synthesize the Ru-W binary oxide catalysts achieving atomic-level uniform metal dispersion via the sol-gel method. The optimized catalyst demonstrates a 20-fold improvement of intrinsic OER activity compared to pristine RuO_2_, which also achieves robust stability for more than 550 h of continuous electrolysis with only 0.014 mV h^−1^ degradation. Electrochemical studies, ex-situ/in-situ near-ambient pressure X-ray photoelectron spectroscopy (NAP-XPS) and density functional theory (DFT) calculations prove that the forming of W-O_bri_-Ru Brønsted acid sites mitigates the too strong proton adsorption energy on O_bri_ of RuO_2_ and enables an easier proton transfer from the oxo-intermediates to the neighboring O_bri_, and thus accelerated the overall acidic OER kinetics. Finally, the universality of such a strategy is confirmed in other Ru-M binary metal oxides (M = Cr, Mo, Nb, Ta, and Ti).

## Results

### Synthesis and characterizations of Ru-W binary oxide catalysts

**Fig. 1:**
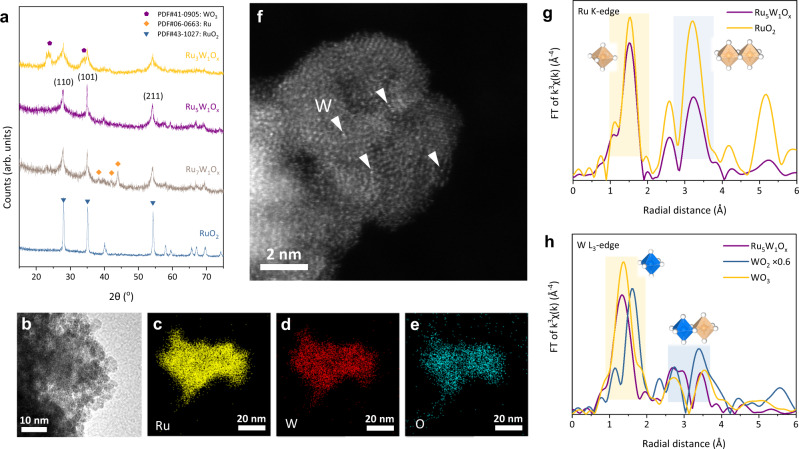
Morphology of Ru-W oxides. **a** The XRD patterns of different catalysts. No peaks from the segregated phases (WO_3_ or metallic Ru) were observed in the pattern of Ru_5_W_1_O_x_. **b** The HR-TEM image of as-prepared Ru_5_W_1_O_x_ catalyst. **c**–**e** The EDX element mapping of Ru, W, and O. **f** The atomic resolution HAADF-STEM image of Ru_5_W_1_O_x_. The bright spots were W atoms. **g**, **h** The *k*^3^-weighted FT-EXAFS spectra of Ru K-edge and W L_3_-edge. The Ru_5_W_1_O_x_ had a shorter W-O distance than other W oxides, indicating a dense packing local structure. While tungsten oxides possess loose packing structures, this shortened W-O distance verified the incorporation of W into rutile RuO_2_ lattice. The orange and blue octahedrons represent RuO_6_ and WO_6_ octahedrons, respectively.

We began with the synthesis of the Ru-W binary oxide catalysts via a modified sol-gel method (see Methods). By adjusting the feed ratio of metal precursors, we finally obtained the rutile Ru_5_W_1_O_x_ catalyst with no obvious phase separation, as shown by X-ray diffraction patterns (XRD) (Fig. 1a). The high-resolution transmission electron microscopy (HR-TEM) showed that the as-prepared catalyst was 4–5 nm nanoparticles (Fig. 1b), with a Brunauer-Emmett-Teller (BET) surface area of 53.86 m^2^ g^−1^ (Supplementary Fig. 2). The element mapping of Energy-dispersive X-ray spectroscopy (EDX) confirmed the homogeneous distribution of Ru, W, and O in the materials (Fig. 1c–e). The spherical aberration corrected high-angle annular dark-field scanning transmission electron microscopy (HAADF-STEM) image (Fig. 1f) showed uniformly dispersed bright spots on the nanoparticle, which came from the atomic dispersion of W atoms into the RuO_2_ lattice. The solid solution feature of Ru_5_W_1_O_x_ was further confirmed by extended X-ray absorption fine structure (EXAFS). According to the Fourier transformed Ru K-edge EXAFS (FT-EXAFS) spectra, the rutile structure was maintained after W incorporation (Fig. 1g). The W atoms demonstrated a completely different coordination environment from common tungsten oxides, with a shorter W-O distance being observed than the WO_3_ standard (Fig. 1f and Supplementary Fig. 3). The wavelet transformed EXAFS spectra of Ru_5_W_1_O_x_ showed a distinct peak at **R** ≈ 3.5 Å, **k** ≈ 11 Å^−1^, which could be attributed to the W-Ru scattering peak (Supplementary Fig. 4). The Raman spectroscopy of Ru_5_W_1_O_x_ demonstrated a diminished rutile *B*_*2g*_ mode (706 cm^−1^) and a peak rising at 771 cm^−1^, confirming the formation of W-O_bri_-Ru structure in the catalyst (Supplementary Fig. 5). All the results above confirmed the atomically dispersed Ru-W solid solution oxide.

### Evaluation of OER performances

**Fig. 2:**
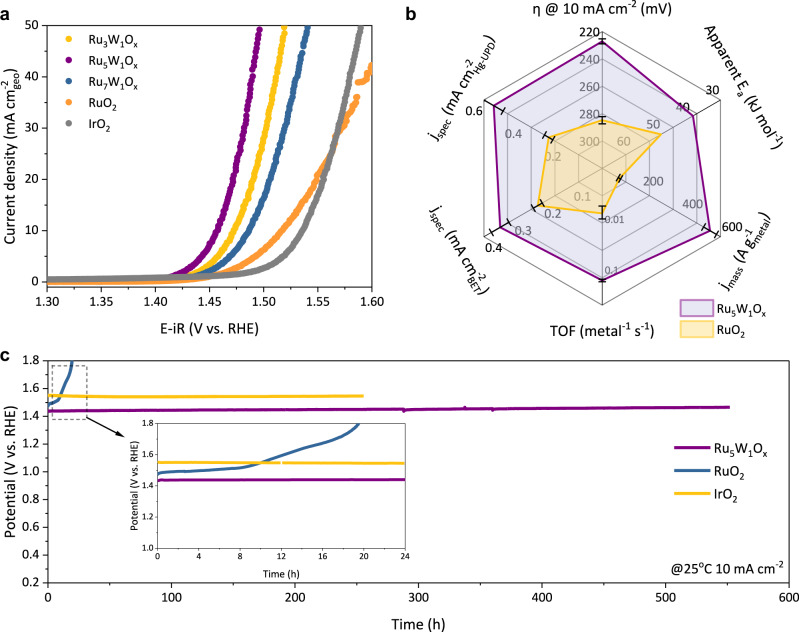
Electrochemical performance of Ru-W oxides. **a** The LSV curves of different catalysts with 95% iR compensation. Scan rate: 5 mV s^−1^. **b** Summary of some major OER performance metrics of Ru_5_W_1_O_x_ and RuO_2_. The specific OER activity (*j*_*spec*_) (normalized by BET surface area and Hg-UPD surface area respectively) was calculated at 1.50 V *vs*. RHE. The apparent activation energy (*E*_*a*_) was calculated by the OER current of 1.50 V *vs*. RHE at different temperatures. The TOF and the mass-specific activity were calculated at *η* = 300 mV based on total metal loading. The error bars are standard deviations of averaging three independent measurements. **c** The stability comparison between Ru_5_W_1_O_x_, RuO_2_, and IrO_2_. The stability of catalysts was evaluated by chronopotentiometry at 10 mA cm^−2^.

We then evaluated the OER performance of Ru_5_W_1_O_x_ in a three-electrode system using 0.5 M H_2_SO_4_ as the electrolyte. All electrode potential was converted to the reversible hydrogen electrode (RHE). The linear sweep voltammetry (LSV) (Fig. 2a) of Ru_5_W_1_O_x_ showed that the catalyst only needed 227 mV overpotential (η) to reach 10 mA cm^−2^ current density −58 mV lower than the commercial nano-RuO_2_ (Sigma-Aldrich, ~20 nm nanoparticles, Supplementary Fig. 6). To systematically compare the OER activity of different catalysts, several other performance metrics were also measured and calculated (Fig. 2b, Supplementary Table 1, and Supplementary Note 1). The OER performance Ru_5_W_1_O_x_ outperformed the nano-RuO_2_ at all six considered dimensions: The mass-specific activity was improved by 8-fold (750 A g_Ru_^−1^ of Ru_5_W_1_O_x_ vs. 87 A g_Ru_^−1^ of RuO_2_, estimated by total Ru loading mass). When calculated by the total metal loading (Ru+W), the mass-specific activity of Ru_5_W_1_O_x_ was 547 A g_metal_^−1^, 6 times higher than the RuO_2_ (Supplementary Fig. 8). The turnover frequency (TOF) of Ru_5_W_1_O_x_ reached 0.163 ± 0.010 s^−1^ (at *η* = 300 mV), which was a 20-fold improvement from the pristine RuO_2_ (0.007 ± 0.002 s^−1^) (Supplementary Fig. 9). The specific activity of Ru_5_W_1_O_x_ was obtained by normalizing the OER current using either the catalyst’s BET surface area or the mercury underpotential deposition (Hg-UPD) determined electrochemical active surface area (ECSA) (Supplementary Fig. 10). Both values surpassed the pristine RuO_2_ by *ca*. 2 times at 1.50 V vs. RHE (Supplementary Fig. 11). The apparent activation energy (*E*_*a*_) was reduced from 42.2 kJ mol^−1^ to 28.4 kJ mol^−1^ after W incorporation (Supplementary Fig. 13). The above results verified that the incorporation of W-O_bri_-Ru Brønsted acid sites improved the OER activity of RuO_2_ both apparently (by the increase of electroactive surface area) and intrinsically (by the increase of activity of per active site), indicating a lower barrier and a different OER mechanism for Ru_5_W_1_O_x_.

We next examined the OER stability of Ru_5_W_1_O_x_ in acid using chronopotentiometry at 10 mA cm^−2^. The catalyst showed no obvious activity decrease in the long-term operation (Fig. 2c). The overpotential was maintained at 235 mV after 550 h of continuous electrolysis, demonstrating a degradation rate of only 0.014 mV h^−1^, which acted as a highly active iridium-free catalyst in long-term operation in acid electrolytes (Supplementary Fig. 14 and Supplementary Table 8). We also performed the same test on the commercial IrO_2_ catalyst (~5 nm, BET surface area 11.98 m^2^ g^−1^, Supplementary Fig. 15), which was highly stable as expected. And the Ru_5_W_1_O_x_ has shown comparable stability to state-of-the-art IrO_2_ catalysts. In the intense cycle test, the Ru_5_W_1_O_x_ could also stay active even after 20,000 CV cycles (Supplementary Fig. 16). While the RuO_2_ showed poor stability in both chronopotentiometry and cycle tests (Supplementary Fig. 17). The morphology and composition of the Ru_5_W_1_O_x_ catalyst didn’t change significantly after electrolysis, as demonstrated by the HR-TEM images and EDX elemental mappings after OER (Supplementary Fig. 18). The in situ EXAFS also indicated that the W-O-Ru structure was retained under OER conditions (Supplementary Fig. 19). At higher electrolysis current densities (100 mA cm^−2^), the stability of Ru_5_W_1_O_x_ was also maintained within a 100-h test (Supplementary Fig. 20). These data showed that the Ru_5_W_1_O_x_ catalyst is a promising candidate for practical application.

### Investigation of deprotonation on bridging oxygen

**Fig. 3:**
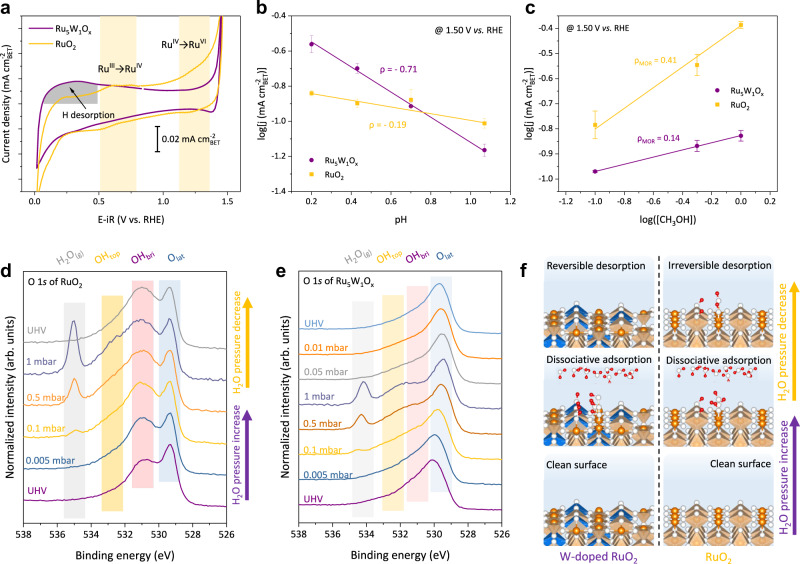
Investigation of deprotonation on bridging oxygen. **a** Typical CV curves of Ru_5_W_1_O_x_ and RuO_2_. Scan rate: 200 mV s^−1^. **b** Logarithm of OER activity at 1.50 V *vs*. RHE as a function of pH. **c** The logarithm plots between MOR current density and concentration of methanol on different catalysts. The current densities in **a–c** were normalized by BET surface area. The error bars in **b**, **c** are standard deviations of averaging three independent measurements. **d, e** The O 1 *s* XPS spectra of RuO_2_ and Ru_5_W_1_O_x_ at different water vapor pressures. Annotations: O_lat_ - lattice oxygen, OH_bri_ – protonated bridging oxygen, OH_top_ – adsorbed or liquid water molecules, H_2_O_(g)_ – gas-phase water molecules. **f** A schematic demonstrated the surface species changes at different water vapor pressures. Orange balls – Ru, Blue balls – W, White balls – O, Red balls – H. The orange and blue octahedrons represent RuO_6_ and WO_6_ octahedrons, respectively.

To investigate the protonation/deprotonation on the catalyst’s surface, we then conducted a series of electrochemical experiments correlated with proton transfer. We first examined the cyclic voltammetry (CV) profile differences between Ru_5_W_1_O_x_ and RuO_2_ (Fig. 3a). The CV of RuO_2_ included two pairs of redox peaks at *ca*. 0.65 V and 1.25 V vs. RHE, which were often attributed to Ru^III^/Ru^IV^ and Ru^IV^/Ru^VI^ surface redox transitions, respectively^22^. In contrast, in Ru_5_W_1_O_x_, the peak at *ca*. 1.25 V became less prominent, while a large plateau located between 0 V and 0.4 V vs. RHE arose instead. This plateau was similar to the hydrogen desorption peak on WO_3_ or Pt electrodes^20,23,24^ (Supplementary Fig. 21), indicating that deprotonation of Ru_5_W_1_O_x_ surface required a much lower potential than RuO_2_. We also checked the electrochemical behavior of Ru_5_W_1_O_x_ in 1 M HClO_4_ (same pH as 0.5 M H_2_SO_4_). No obvious electrolyte effect could be observed, which indicates that the adsorption of sulfate will not interfere with the surface chemistry of Ru, different from the Ir-based catalysts^25,26^ (Supplementary Fig. 22).

**Fig. 4:**
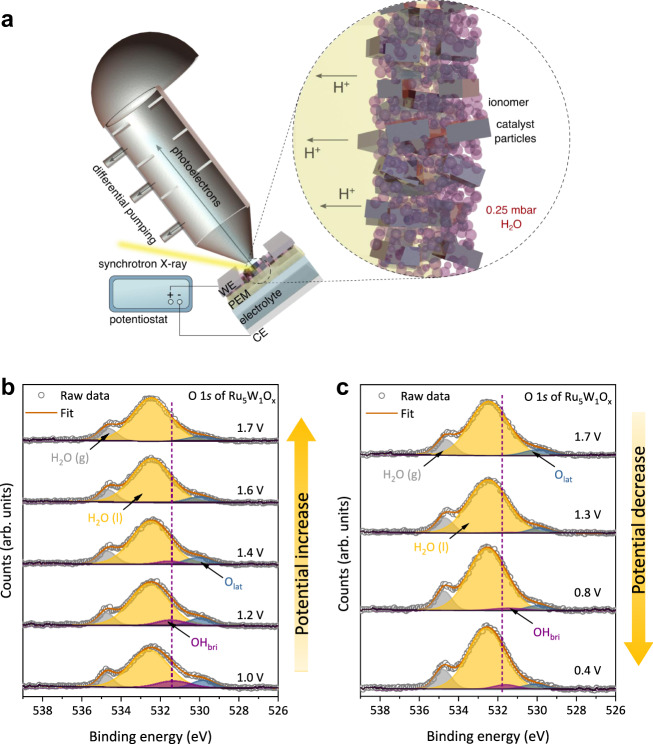
In situ spectroscopic investigation of OER process. **a** A schematic of the in situ electrochemical measurements. The zoom area illustrates the major components at the measured electrochemical interface. **b, c** In situ electrochemical O 1 *s* XPS spectra of Ru_5_W_1_O_x_. As the potential increased, the OH_bri_ peak decreased accordingly, indicating the deprotonation of W-OH_bri_-Ru sites during OER. The O_bri_ sites were re-protonated when decreasing the potential. The binding energy of all spectra was calibrated according to the Au 4 *f* peak at 84.0 eV. The pressure of the XPS chamber was maintained at 0.25 mbar by injecting water vapor.

We then examined the correlation between electrolyte pH and OER activity on different catalysts at the RHE scale^27^ (Supplementary Fig. 23). As shown in Fig. 4b, Ru_5_W_1_O_x_ demonstrated pH-dependent OER activity, with a reaction order (ρ) of −0.71. While for RuO_2_, the ρ is only −0.19, demonstrating a weak pH-dependence of OER activity, coinciding with the previous report^22^. This pH-dependent activity difference could be elaborated by the acidity of bridging hydroxyl: the proton dissociation constant (pK_a_) of Ru-OH_bri_-Ru >> pH and the O_bri_ sites of RuO_2_ were saturated by protons within the experimental pH range. Whereas the W-OH_bri_-Ru showed a stronger Brønsted acidity (pK_a_ of OH_bri_ close to pH), leading to a sensitive pH dependence of OER activity. Further verification of the Brønsted acidity of W-OH_bri_-Ru sites was given by the ^1^H solid-state nuclear magnetic resonant (^1^H-NMR) spectrum, in which a split peak was observed indicating the formation of different OH_bri_ sites^28,29^ (Supplementary Fig. 24).

We next measured the surface OH* coverage on both catalysts using methanol as a molecular probe^30,31^. The methanol oxidation reaction (MOR) has a well-established mechanism that methanol molecules tend to nucleophilically attack the electrophilic OH*, so MOR will be more active on an OH* dominated surface^31^. We measured the reaction order of MOR on both catalysts (Fig. 3c and Supplementary Fig. 25) and found that Ru_5_W_1_O_x_ is inert toward MOR, demonstrating a low surface OH* coverage. While RuO_2_ showed higher MOR activity, indicating the RuO_2_ surface was dominated by OH*. The above results verified that the deprotonation was easier on Ru_5_W_1_O_x_ under applied potentials.

We finally analyzed the steady-state Tafel plots to study the apparent OER kinetics of different catalysts (Supplementary Fig. 26). The RuO_2_ showed a 54 mV per decade (mV dec^−1^) Tafel slope, suggesting that the reaction has a one-electron transfer electrochemical pre-equilibrium step (PES) followed by a pure chemical rate-determining step (RDS) with no electron transfer^7,21,22^. While Ru_5_W_1_O_x_ showed a 42 mV dec^−1^ slope. It corresponds to a one-electron transfer electrochemical PES followed by another one-electron transfer electrochemical RDS. We attributed these differences to the different proton binding energy on O_bri_. Considering the BOAD pathway, protonated Ru-OH_bri_-Ru might inhibit the chemical proton transfer from the OER intermediates (OH* or OOH*) to the O_bri_, while the less protonated W-O_bri_-Ru sites could favor the proton transfer to the O_bri_, thus shifting the RDS. The detailed deduction and discussion of the Tafel slope refer to Supplementary Note 2.

### Surface oxygen species study by NAP-XPS

To obtain further insights into the deprotonation process on O_bri_, we then carried out NAP-XPS measurements under varied water vapor pressure (Supplementary Note 3). As shown in Fig. 3d, e, four different oxygen species were distinguished by O 1 *s* XPS spectra: the lattice oxygen (O_lat_) at *ca*. 530 eV, the protonated bridging oxygen (OH_bri_) at *ca*. 531 eV, molecularly adsorbed water/hydroxyl (OH_top_) adsorbed on the coordinatively unsaturated Ru sites (Ru_CUS_) at *ca*. 533 eV and the gas phase water molecules (H_2_O_(g)_) at 534-535 eV^32^. Under ultra-high vacuum (UHV), RuO_2_ showed a more than 3 times higher ratio of OH_bri_ species than Ru_5_W_1_O_x_ (Supplementary Fig. 29, Supplementary Table 3 and 4). With the increase in water vapor pressure, the ratio of OH_bri_ in RuO_2_ increased accordingly (Fig. 3d), which was contributed by the dissociative adsorption of water molecules on the RuO_2_ surface^32^ (Supplementary Fig. 30). However, when reduced the pressure to UHV, the OH_bri_ ratio did not decrease accordingly, which verified the strong proton adsorption nature of O_bri_ in the Ru-O_bri_-Ru structure.

In contrast, several different features were observed in the O 1 *s* XPS spectra of Ru_5_W_1_O_x_ (Fig. 3e). Firstly, the O_lat_ peak positively shifted by ~0.5 eV, again proving the formation of Ru-W oxide solid solution^33^. The most prominent difference between Ru_5_W_1_O_x_ and RuO_2_ lies in the OH_bri_ transition. The OH_bri_ intensity did not change significantly along with the vapor pressure change. Instead, the O_lat_ shifted to lower binding energy, accompanied by valence changing of W from W^6+^ to W^5+^, as observed in W 4 *f* XPS spectra (Supplementary Fig. 31 and Supplementary Table 5). These peak shifts were reversible when sequentially reducing the pressure back to UHV (Fig. 3e). The above results displayed a scenario that the deprotonation of water molecules (or oxo-intermediates during OER) was faster and more reversible in Ru_5_W_1_O_x_ than that in RuO_2_ (Fig. 3f). Detailed discussions refer to Supplementary Note 3.

The deprotonation process on Ru_5_W_1_O_x_ was further monitored using an in-situ electrochemical NAP-XPS setup (Fig. 4a and Supplementary Fig. 32). As the potential increased, the OH_bri_ peak decreased accordingly, demonstrating a potential-dependent deprotonation scenario (Fig. 4b). When reducing the applied potential, the O_bri_ protonated and formed OH_bri_ again (Fig. 4c), providing evidence of the reversible protonation/deprotonation nature of W-O_bri_-Ru sites. On the contrary, due to the strong interaction between water and Ru-O_bri_-Ru, the RuO_2_ surface was covered by condensed water or OH_top_ species and the deprotonation of OH_bri_ could hardly be observed upon applying electrode potentials (Supplementary Fig. 35). The deprotonation process can also be verified by the in situ Raman spectroscopy (Supplementary Fig. 38). In Ru_5_W_1_O_x_, the peak at *ca*. 880 cm^−1^ decreased along with the potential increase, indicating the deprotonation of W-OH_bri_-Ru along with the potential increase. Detailed discussions on the in situ electrochemical XPS experiment refer to Supplementary Note 4.

### Theoretical investigation of the deprotonation energetics on Brønsted acid sites

**Fig. 5:**
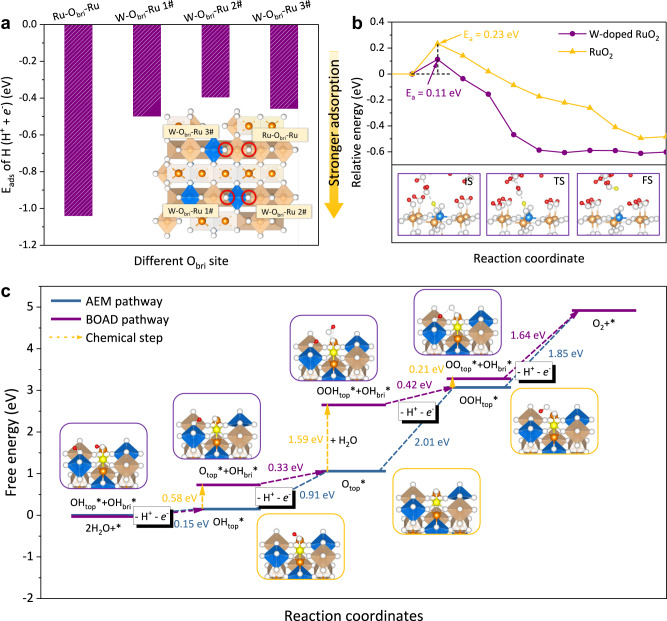
DFT investigation of hydrogen adsorption on Ru-W binary oxides. **a** The H atom adsorption energy on different surface O_bri_ sites. Inset: Schematic of different O_bri_ sites on the W-doped RuO_2_. **b** The kinetic barrier of the deprotonation of OH_bri_ on different catalysts with solvent. Insets: The snapshots of the initial state (IS), transition state (TS), and final state (FS) on W-doped RuO_2_. **c** The free energy diagram of W-RuO_2_ with different OER pathways. The active Ru site is marked yellow. Orange balls – Ru, Blue balls – W, White balls – O, Red balls – H. The orange and blue octahedrons represent RuO_6_ and WO_6_ octahedrons, respectively.

To further understand the relationship between the Brønsted acidity of O_bri_ and OER activity. We investigated the effect of introducing Brønsted acid sites into RuO_2_ using DFT calculations. We inserted the WO_6_ octahedrons into the stable RuO_2_ (110) facet and constructed two types of O_bri_ sites: Ru-O_bri_-Ru and W-O_bri_-Ru^34^ (Fig. 5a inset). We then examined the adsorption energy (E_ads_) of hydrogen atoms (by assuming the energy of H^+^ + *e*^−^ as the energy of 1/2 H_2_ molecule) on different O_bri_ sites. The Ru-O_bri_-Ru demonstrated strong adsorption energy of H with an E_ads_ of −1.04 eV, while the W-O_bri_-Ru showed mild H adsorption energy ranging from −0.39 eV to −0.50 eV (Fig. 5a). This indicated that protons tended to spontaneously adsorb onto Ru-O_bri_-Ru sites in acidic electrolytes. Thus extra energy input was needed to deprotonate the proton-saturated Ru-OH_bri_-Ru sites under OER conditions for pristine RuO_2_. In contrast, the low H adsorption energy on W-O_bri_-Ru enables much easier deprotonation of the OH_bri_ (a stronger Brønsted acidity). Since the deprotonation of OH_bri_ was regarded as the rate-limiting step in Ru-based catalysts at low overpotential^8,35^, we further calculated the kinetic barrier of the deprotonation on different O_bri_ sites considering the effect of solvent (Supplementary Note 5, Supplementary Fig. 43 and 44). The W-OH_bri_-Ru model showed a lower barrier of deprotonation compared with Ru-OH_bri_-Ru, indicating a faster deprotonation process on W-OH_bri_-Ru (Fig. 5b). All these DFT results coincided with the electrochemical and XPS measurements, which well explained how the Brønsted acid sites promoted the OER kinetics. To further understand the pH-dependent activity of the Ru-W catalyst, we checked the E_ads_ of protons on W-O_bri_-Ru with all Ru-O_bri_-Ru saturated by protons (Supplementary Fig. 40). The E_ads_ of protons kept reducing along with the increase of hydrogen coverage and finally reached nearly thermal-neutral adsorption energy (−0.06 eV), indicating high proton mobility of W-doped RuO_2_ in strong acidic electrolytes.

By integrating the above electrochemical, spectroscopic, and theoretical results, we finally proposed an OER pathway including BOAD steps on the W-doped RuO_2_ catalyst (Fig. 5c). In this mechanism, the O_bri_ played a critical role in water dissociation and oxo-intermediates deprotonation. At each step, the adsorbed oxo-intermediate (or water molecule) first chemically transfers a proton to the neighboring W-O_bri_-Ru site, afterwards, the OH_bri_ deprotonates accompanying an electron transfer. We calculated the thermodynamic OER overpotential of Ru_5_W_1_O_x_ based on both the BOAD pathway and conventional adsorbates evolution mechanism (AEM) pathway using DFT^9^. The BOAD pathway showed an overpotential of 0.41 V while the AEM showed an overpotential of 0.78 V–a 0.37 V improvement by the BOAD mechanism.

### The universality of BOAD steps

**Fig. 6:**
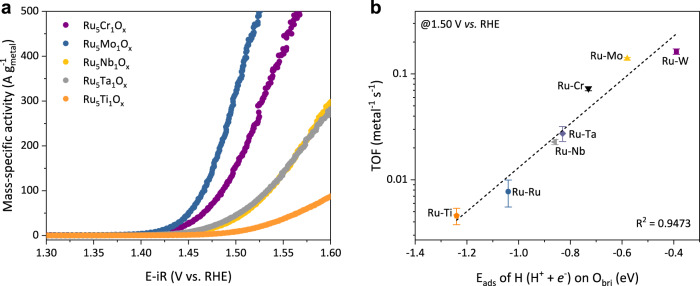
The universality of the BOAD steps. **a** The mass-specific activity of different catalysts in 0.5 M H_2_SO_4_ based on total metal loading. Scan rate: 5 mV s^−1^. **b** The TOF value of different catalysts (regarding all metal atoms were active sites) as a function of H adsorption energy on O_bri_ sites.

To extend our strategy of regulating Brønsted acidity of O_bri_ in acidic water oxidation, we further replaced W with other metals (M = Cr, Mo, Nb, Ta, and Ti), which are often used as Brønsted acids or bases, to form rutile-type oxides and examined their OER performances (Fig. 6a). The hydrogen adsorption energy of M-O_bri_-Ru sites was also calculated using DFT. We found a linear relationship between the OER activity (represented by the TOF of Ru atoms) and the E_ads_ of H atoms on O_bri_ sites (Fig. 6b). The results showed that increasing the acidity of the O_bri_ site on RuO_2_ could lead to easier deprotonation and accelerate the BOAD process, which confirms the validity of our modulating strategy.

In summary, in this work, we demonstrated a strategy to modify the Brønsted acidity of bridging oxygen sites in RuO_2_ to improve acidic water oxidation. The incorporation of Brønsted acid sites (e.g. WO_x_) could optimize the proton adsorption energy of bridging oxygen sites. The electrochemical, in-situ and ex-situ X-ray spectroscopic and theoretical studies proved that: these W-O_bri_-Ru bridging oxygen sites increased the mobility of protons on the catalyst surface and led to a fast bridging-oxygen-assisted deprotonation process, thus accelerating the OER kinetics. This strategy was proved to be universal in other Ru-M binary metal oxides (M = Cr, Mo, Nb, Ta, and Ti), and all catalysts demonstrated an excellent linear relationship between the OER activity and the E_ads_ of protons on O_bri_ sites. This work provides new insights into the OER mechanism and broadens the designing principles for new high-performance electrocatalysts.

## Methods

### Computational methods

All the calculations were performed by periodic DFT with the Vienna Ab-initio Simulation Package (VASP) code^36^. The projector augmented wave (PAW) method was used to describe the interaction between the atomic cores and electrons^37,38^. The kinetic energy cut-off for the plane-wave expansion was set to 400 eV, and the Brillouin-zone integrations for the adsorption model were sampled using a (3 × 3 × 1) Monkhorst–Pack mesh^39^. The generalized gradient approximation (GGA) with PBE functional was used^40^. The convergence thresholds of the energy change and the maximum force for the geometry optimizations were set to 10^−6^ eV and 0.03 eV/Å, respectively. A vacuum of 15 Å in the z-direction was employed to avoid the interactions between periodic images.

For the chemisorption on W modified RuO_2_ (110) surface, many possible adsorption configurations were explored, and the thermodynamic stability of different structures was determined by the adsorption energy (ΔE_ads_) that was defined as,

1 $$\triangle {E}_{{ads}}={E}_{*M}-{E}_{*}-{E}_{M}$$

where E_*M_ and E_*_ represent the total energies of catalyst surface with and without adsorbate, respectively; *E*_*M*_ is the total energy of adsorbate. All of them are available from the DFT calculation.

The computational hydrogen electrode (CHE) model^41^ was employed to calculate the Gibbs free energy change (Δ*G*) for each elementary reaction step and construct the free energy diagram for the OER. The Δ*G* was computed using the following:

2 $$\triangle G=\triangle E+\triangle {ZPE}-T\triangle S+\triangle {G}_{U}+\triangle {G}_{{pH}}$$

where Δ*E* is the reaction energy between the initial state and the final state of the elementary reaction, which is available from DFT total energy; the correction of zero-point energy (Δ*ZPE*) and entropy at *T* = 298.15 K (*T*Δ*S*) can be obtained from vibrational frequency calculations. Δ*G*_*U*_ = *nU*, where *n* and *U* stand for the number of electrons transferred and the applied electrode potential, respectively. Δ*G*_*pH*_ = *k*_*B*_*T* × *ln10* × *pH*, where *k*_*B*_ is the Boltzmann constant. The free energy change between 1/2 H_2_ and H^+^ + *e*^−^ will be zero at the potential of 0 V and 1/2 *G*_*(H2)*_ can be equal to the free energy of proton and electron.

To simulate the interaction at the water/(W-doped)RuO_2_ interface, we used 18 explicit water molecules (6 layers) on a 2 × 1 RuO_2_ surface slab (3 layers) with an area of 6.28 × 6.42 Å^2^. The simulation box is 28 Å along the z-axis. The initial structure of the water box is based on the density of the solvent^42,43^ (as shown in Supplementary Fig. 43). To equilibrate the waters interacting with the interface, we carried out 850 steps (time step is 1 fs) of ab initio molecular dynamics (AIMD) simulation at 298 K^44^. The temperature and potential energies during the AIMD simulation are shown in Supplementary Fig. 44. To calculate deprotonation barriers of adsorbed H, we made use of the climbing image nudged elastic band (CI-NEB) method^45^ based on the established models.

### Synthesis of catalysts

The Ru-W binary oxide catalysts were synthesized by a sol-gel method. In a typical procedure, 0.75 mmol ruthenium trichloride hydrate (RuCl_3_·xH_2_O, Sigma-Aldrich) and 0.15 mmol tungsten hexachloride (WCl_6_, Sigma-Aldrich) were first dissolved in 3 mL N, N-dimethylformamide (DMF) and cooled in a refrigerator for 2 h. Then 200 μL of deionized water was added. In the meantime, 500 μL propylene oxide (Sigma-Aldrich) was dropwise added into the solution using a syringe pump under stirring. The solution was then placed and aged overnight before the reaction was quenched by adding acetone. The formed precipitations were washed with acetone 3 times and dried in the vacuum. The dried powder was then grounded and annealed at 500 °C for 1 h to obtain the final catalyst.

To synthesize other reference catalysts, the same procedure was used by adjusting the ratio of precursors (the total amount of metal precursors was controlled at 0.9 mmol) or changing the metal precursors. The synthesis of Ru-M (M = Cr, Mo, Nb, Ta, and Ti) followed the same procedure with Ru_5_W_1_O_x_. Chromium chloride hexahydrate (CrCl_3_·6H_2_O), molybdenum chloride (MoCl_5_), niobium chloride (NbCl_5_), tantalic chloride (TaCl_5_), and titanium tetrachloride (TiCl_4_) (all purchased from Sigma-Aldrich) were used as metal precursors. The RuO_2_ nanoparticles (~25 nm) were purchased from Sigma-Aldrich without further treatment. The commercial IrO_2_ nanoparticles (~20 nm) were purchased from PERIC Inc.

### Materials characterizations

The X-ray diffraction (XRD) patterns of prepared catalysts were measured by a Bruker D8A diffractometer. The Brunauer-Emmett-Teller surface area of the catalysts was obtained by a Quantachrome Autosorb-iQ analyzer. An FEI Tecnai G20 transmission electron microscope (TEM) was used to obtain the high-resolution TEM (HR-TEM) images and corresponding energy-dispersive X-ray spectroscopy (EDX) elemental mapping were from with an Oxford energy disperse spectrometer. The spherical aberration corrected high-angle annular dark-field scanning transmission electron microscopy (HAADF-STEM) images were obtained by a Titan G2 300 kV TEM (Thermo Fisher Scientific).

The hard (Ru K-edge and W L_3_-edge) X-ray Absorption spectroscopies (XAS) measurements were carried out at the 1W1B beamline of the Beijing Synchrotron Radiation Facility (BSRF), respectively. The XAS data were processed, normalized, and fitted using the Demeter software package embedded with FEFF 8.5 codes^46^. The wavelet transformation of EXAFS spectra was performed by WTEXAFS software^47^.

The ^1^H solid-state nuclear magnetic resonance (^1^H-NMR) spectroscopy was performed on a Bruker 400WB AVANCE III spectrometer. The catalysts powder was dehydrated at 300 °C in the air for 2 h before measurements.

### Electrochemistry

The evaluation of electrochemical performance was carried out in a three-electrode system. All electrolytes were purged by argon to remove the dissolved oxygen during measurements. To prepare the working electrode (WE), 5 mg catalyst powder, 2 mg carbon black (XC-72), 980 μL mixed solution (water: ethanol = 5:1, v/v), and 20 μL Nafion solution (5 wt%, Sigma-Aldrich) were mixed and sonicated to form a homogeneous ink. Then, 4.5 μL ink (catalyst loading = 0.0225 mg) was drop-cast onto a clean glassy carbon rotating disk electrode (RDE, Autolab, 3 mm in diameter) and dried at room condition. All electrochemical measurements were carried out by a Metrohm Autolab PGSTAT204 potentiostat. A saturated mercurous sulfate electrode (MSE, *E*_*0*_ = 0.652 V vs. RHE) was used as the reference electrode (RE) and a Pt foil was used as the counter electrode (CE). The measured potential was calibrated to the RHE scale according to:

3 $${E}_{{RHE}}={E}_{H{g}_{2}S{O}_{4}} \,+\, 0.652 \,+\, 0.0591\times {pH}$$

To evaluate the OER activity of different catalysts, the WEs were first performed 10 CV cycles between 0.95 to 1.50 V *vs*. RHE (before iR-correction) at a 50 mV s^−1^ scan rate to clean and stabilize the surface, then followed with an LSV scan from 0.95 to 1.65 V at 5 mV s^−1^ scan rate and 2500 rpm rotation speed. For the pH-dependent activity measurement, 0.05, 0.1, 0.25 and 0.5 M H_2_SO_4_ solution (pH = 1.1, 0.7, 0.4, 0.2, respectively. Measured by a Horiba D-71 pH meter) was used as electrolyte without adding buffer salt. The methanol oxidation was measured in 0.5 M H_2_SO_4_ containing different concentrations of methanol. The steady-state Tafel slope was measured by elevating the potential from 1.25 to 1.75 V vs. RHE by 20 mV each step. Each step was maintained for 10 s. The uncompensated solution resistances (R_Ω_) were measured by extrapolating the electrochemical impedance semi-circle to the high-frequency end, which was *ca*. 7 Ω for each electrode in 0.5 M H_2_SO_4_.

The turnover frequency values were calculated according to the following equation:

4 $${TOF} \,=\, \frac{j\times A\times \eta }{4\times e\times n}$$

where *j* is the current density at 1.53 V *vs*. RHE after 95% iR compensation, *A* is the geometric area of GCE (0.0706 cm^2^), *η* is the Faradic efficiency and *e* is the charge of an electron (1.602 × 10^−19^ C) and *n* is the number of active sites.

The active site number *n* was determined by assuming all Ru atoms (or all metal atoms) as active sites (underestimating case), according to the following equation:

5 $${n}_{{mass}} \,=\, \frac{{m}_{{loading}} \, \times \, {N}_{A}}{{Mw}} \, \times \, {n}_{{metal}}$$

where *m*_*loading*_ is the loading mass of the catalyst. *N*_*A*_ is Avogadro’s constant (6.022 × 10^23^ mol^−1^), *Mw* is the molecular weight of catalysts (estimated by the molecular formula Ru_5_W_1_O_13_ for Ru_5_W_1_O_x_) and *n*_*metal*_ is the number of Ru atoms or metal atoms per molar of catalysts.

The stability measurements were carried out by air-brush spraying the catalysts powder onto the carbon paper (TGP-H-060, Toray). The catalyst loading was *ca*. 1.5 mg cm^−2^. The electrolysis cell was kept in a 25 °C constant temperature water bath. 100 μL of water was added to the cell every four days to keep the concentration of the electrolyte constant.

### Evaluation of the electrochemical active surface area (ECSA)

In this paper, we used mercury underpotential deposition^48,49^ (Hg-UPD) and electrochemical double-layer capacitance (*C*_*dl*_) to evaluate the ECSA of different catalysts. To prepare the WE, 2 mg catalysts powder and 1 mg XC-72 carbon black were sonicated in 2 mL water/ethanol mix solution containing 20 μL Nafion solution and 3 μL obtained ink was drop-casted on the RDE. The catalyst loading was 42.5 μg cm^−2^.

For the Hg-UPD method, the fresh electrode was first cycled in Ar-purged 0.1 M HClO_4_ at −0.15 to +0.65 V vs. MSE to obtain the background (50 mV s^−1^, 1600 rpm). Then, the same electrode was moved to an Ar-purged 0.1 M HClO_4_ electrolyte containing 1 mM Hg(NO_3_)_2_ (Alfa Aesar) and cycled under the same condition. The current difference of the cathodic scans between the Hg-containing solution and blank background was integrated to calculate the amount of Hg_upd_. A coulombic charge of 138.6 μC cm^−2^ was used as a factor to obtain the Hg_upd_-ECSA values.

For the double-layer capacitance method, the C_dl_ values were obtained by conducting CV cycles at various scan rates from 20 mV s^−1^ up to 200 mV s^−1^ in Ar-purged 0.5 M H_2_SO_4_. The CVs were scanned between 0.20 and 0.30 V vs. MSE. The cathodic and anodic charging currents measured at 0.25 V vs. MSE (close the open circuit potential) were plotted as a function of scan rate. The average slope of the anodic and cathodic plot is the *C*_*dl*_ value. A general specific capacitance (*C*_*s*_) of 35 μF cm^−2^ was used to calculate the *C*_*dl*_-derived ECSA^50^.

### Near-ambient pressure X-ray photoelectron spectroscopy (NAP-XPS)

The AP-XPS spectra were measured at the BL02B01 beamline of Shanghai Synchrotron Radiation Facility^51^ (SSRF). The incident photon energy was set to 735 eV to distinguish the surface species. To measure the adsorption isotherm of water vapor, the powder catalysts were first tableted and mounted into the analysis chamber. Before the measurements, the catalysts were first heated to 250 °C under 0.1 mbar O_2_ atmosphere for 30 min to remove the adsorbed water and carbon species. Then, the chamber was pumped back to UHV and returned to room temperature. Ru 3*d*, O 1 *s*, and W 4 *f* XPS spectra were collected at this stage and regarded as the initial state. In the following experiments, different amount of water vapor was injected into the chamber successively, and the XPS spectra were measured under different conditions. For each catalyst, two independent measurements were performed to ensure the validity of the results. Other details of the NAP-XPS experiment are described in Supplementary Note 2.

### In situ electrochemical XPS

The in situ electrochemical XPS experiments were also carried out at the BL02B01 of SSRF, using a homemade electrochemical cell. The design of the electrochemical cell was similar to the cell reported by Falling et al.^52^. The cell was equipped with a gold-coated titanium lid as the WE and a Pt foil as the CE and RE. A Nafion 117 proton-exchange membrane (PEM) was used to seal the electrolyte from the vacuum. To prepare the sample, the interested catalysts were first spray-coated onto the PEM and hot-pressed at 140 °C, then boiled the catalyst-coated membrane in 0.5 M H_2_SO_4_ and deionized water to remove the impurities. During the measurements, the cell was filled with 0.05 M H_2_SO_4_ as the cathodic electrolyte (the anodic electrolyte was the PEM). The pressure of the XPS chamber was maintained at 0.25 mbar by injecting water vapor to relieve the evaporation of electrolytes and provide reactant. The incident photon energy was set to 735 eV to distinguish the surface species. A Biologic SP-200 potentiostat was used to apply potentials. The CE of the cell was grounded to the electron energy analyzer so that the potential of the WE can be directly controlled by the potentiostat. At each potential, an Au 4*f* spectrum on the lid was measured to calibrate the binding energy. Other details of the in situ XPS experiment are described in Supplementary Note 3.

### In situ Raman spectroscopy

The Raman spectra of the powder catalysts were measured either on a Horiba XploRA or a Renishaw In Via Qontor Raman spectrometer. The in situ electrochemical Raman spectroscopy was performed on a Horiba XploRA Raman spectrometer equipped with a 60× waterproof objective and a 638 nm laser. In the in situ measurements, a homemade electrochemical cell, equipped with a saturated Ag/AgCl reference electrode and a Pt wire counter electrode, was used. The spectra were collected at the steady-state under different applied potentials. Each spectrum was integrated for 10 s and averaged by two exposures.

### Reporting summary

Further information on research design is available in the Nature Research Reporting Summary linked to this article.

## Supplementary information

Suppelementary information

Peer Review File

Reporting Summary

## Data Availability

The authors declare that all data supporting the results of this study are available within the paper and its supplementary information files or from the corresponding author upon reasonable request. The electrochemical data of OER performances is provided as the Source Data in this paper. Source data are provided with this paper.

## Source data

Source Data
