# Supplementary material for: Introducing Brønsted acid sites to accelerate the bridging-oxygen-assisted deprotonation in acidic water oxidation
Source: Nat Commun. 2022 Aug 18;13:4871. doi: 10.1038/s41467-022-32581-w (PMC9388623; doi:10.1038/s41467-022-32581-w)
Supplement: Supplementary file 1 — Suppelementary information [file 41467_2022_32581_MOESM1_ESM.pdf]

Supplementary Information for

Introducing Brønsted acid sites to accelerate the bridging-oxygen-assisted deprotonation in acidic water oxidation

Yunzhou Wen[1]†, Cheng Liu[2]†, Rui Huang[1], Hui Zhang[3], Xiaobao Li[3], F. Pelayo García de Arquer[4], Zhi Liu[3,5], Youyong Li[2]* and Bo Zhang[1]*

[1]*State Key Laboratory of Molecular Engineering of Polymers, Department of Macromolecular Science, Fudan University, Shanghai 200438, China.*

[2]*Institute of Functional Nano & Soft Materials (FUNSOM) and Jiangsu Key Laboratory for Carbon-Based Functional Materials & Devices, Soochow University, Suzhou 215123, China.*

[3]*State Key Laboratory of Functional Materials for Informatics, Shanghai Institute of Microsystem and Information Technology, Chinese Academy of Sciences, Shanghai 200050, China.*

[4]*ICFO - Institut de Ciències Fotòniques, The Barcelona Institute of Science and Technology, Barcelona 08860, Spain.*

[5]*School of Physical Science and Technology and Center for Transformative Science, ShanghaiTech University, Shanghai 201210, China.*

†*These authors contributed equally to this work.*

**Correspondence and requests for materials should be addressed to Youyong Li (yyli@suda.edu.cn) (Y.L.) and Bo Zhang (bozhang@fudan.edu.cn) (B.Z.).*

Contents

Supplementary Notes **3**
Supplementary Note 1: Determination of the electrochemical active surface area ... 3
Supplementary Note 2: Tafel slope and OER kinetic analysis 4
Supplementary Note 3: Analysis of NAP-XPS data 5
Supplementary Note 4: In-situ electrochemical XPS 6
Supplementary Note 5: The modeling of electrolyte/catalyst interface 7
Supplementary Figures **9**
Supplementary Tables **31**
Supplementary References **39**

Supplementary Notes

Supplementary Note 1: Determination of the electrochemical active surface area

To comprehensively determine the specific OER activity of the Ru-W catalyst, we adopted three different methods to calculate the electrochemical active surface area (ECSA) of the catalysts and discussed the pros and cons of different methods.

(1) The BET surface area. The first method is using the BET surface area of the total loaded catalyst powder. For nano-catalysts, the catalyst particles are severely agglomerated. The morphology of the catalyst film on the GCE is similar to the agglomerated catalyst powder. So the BET surface area of the catalyst powder can partly reflect the electroactive area of the catalyst. However, the measurement of BET surface area is based on the physical adsorption of N_2 molecules, which is different from the chemical adsorption of reactants, so it cannot fully represent the actual ECSA[1]. It is an overestimated method of the electroactive area.

(2) The Hg-UPD surface area. The second method to calculate the ECSA is based on the mercury underpotential deposition (Hg-UPD). This was obtained by performing cyclic voltammetry in 0.1 M $HClO_4$ containing 1 mM $Hg(NO_3)_2$. The current difference of the cathodic scans between Hg-containing solution and blank background was integrated to calculate the amount of Hg_{upd}. A coulombic charge of 138.6 μC cm^{-2} was used as a factor to obtain the ECSA values. As can be seen in Supplementary Figure 10, the underpotential deposition of Hg on the catalyst surface started at *ca.* 0.35 V *vs.* mercurous sulfate electrode (MSE). Two deposition plateaus appeared on $Ru_5W_1O_x$ while only one on the RuO_2, indicating the presence of different surface sites after W doping. The underpotential deposition method is relatively accurate, which showed lower ECSA values compared to the BET surface area (Supplementary Table 2).

(3) The electrochemical double-layer capacitance. The third method to calculate the ECSA is using electrochemical double-layer capacitance (C_{dl}). The typical method to measure the C_{dl} is scanning CVs in non-Faradic regions at different scan rates. Here we chose a 0.1 V potential window around the open circuit potential (OCP) to conduct the measurement (typically centered at 0.25 V vs. MSE, Supplementary Figure 12). The ECSA was obtained by dividing the C_{dl} with a factor of 35 μF cm^{-2}, according to the previous report[2]. The general trend of the C_{dl}-derived ECSA was the same as the BET and Hg-UPD methods, but the absolute value was higher. Measuring C_{dl} in the non-Faradic region is one of the most widely used methods in evaluating the ECSA of oxides. However, the measured C_{dl} of this method varied from different potential ranges, which could result in uncertainty of ECSA evaluation. Besides, other possible contributions to the measured capacitance have not been taken into account in this method, e.g. the pseudocapacitance in Ru/Ir oxides. Some oxides may experience phase transition before OER onset (e.g. NiO_x), and this could lead to significant conductivity change of catalyst film, which also causes uncertainty of C_{dl} measurements[3].

We summarized the ECSA value obtained by different methods in Supplementary Table 2. All three methods indicated that the $Ru_5W_1O_x$ had a higher electroactive area than the model catalysts. After normalizing the OER current using these different ECSA values, the $Ru_5W_1O_x$ still outperformed the pristine RuO_2 (Supplementary Figure 11). We concluded that the overall improvement of the OER activity in $Ru_5W_1O_x$ was contributed by both the surface area effect and the intrinsic activity of active sites.

Supplementary Note 2: Tafel slope and OER kinetic analysis

The Tafel slope analysis is a classic approach to obtaining the kinetic insights of electrochemical reactions. In this work, we noticed that $Ru_5W_1O_x$ and RuO_2 had different Tafel behavior (Supplementary Figure 26). Both $Ru_5W_1O_x$ and RuO_2 showed two-step Tafel lines. For $Ru_5W_1O_x$, the Tafel slope was 42 mV dec^{-1} at the low overpotential region and 88 mV dec^{-1} at the high overpotential region. For RuO_2, the Tafel slope was 54 mV dec^{-1} at the low overpotential region and 125 mV dec^{-1} at the high overpotential region. Here we focused on the first Tafel slope. Generally, the key process of OER involves a pre-equilibrium step (PES) and a rate-determining step (RDS), which determines the Tafel slopes of the catalysts[4-6]. For each linear region, the Tafel slope *b* can be divided into:

$$b = \frac{d\eta}{\mathrm{dlog}(j)} = \frac{k_B T ln(10)}{(\gamma + n_{rds}\alpha_{rds})e} = \frac{59}{\gamma + n_{rds}\alpha_{rds}} \ (mVdec^{-1}) \qquad (1)$$

In which γ = number of electrons transferred to reach the pre-equilibrium surface, n_{rds} can be either 0 or 1 (0 for a chemical step and 1 for an electrochemical step), α_{rds} is the transfer coefficient of the RDS, typically equal to 0.5 for symmetry potential energy surface. The apparent transfer coefficient (β) can be defined as $\beta = \gamma + n_{rds}\alpha_{rds}$. $k_B T ln(10)/e$ is 59 mV at 298 K. k_B is the Boltzmann's constant, T is the absolute temperature, e is the elementary charge. The change in the experimental Tafel slope is associated with a change in the RDS or the change in the pre-equilibrium surface. In this work, we used the *free energy surface approach* to deduce the OER mechanism[5,7], which is a combination of the experimental Tafel slope and the theoretical *ab initio* calculations.

For $Ru_5W_1O_x$, at the first Tafel region, the b = 42 mV dec^{-1}, β = 1.40, so the γ = 1, n_{rds} = 1, α_{rds} = 0.40. The Tafel slope was determined by an electrochemical PES and an electrochemical RDS. We then calculated the surface Pourbaix diagram of Ru-W binary oxide using DFT (Supplementary Figure 41 and Supplementary Figure 42). We noted that due to the weak proton adsorption of W-O_{bri}-Ru, the surface was fully deprotonated and form an O-terminated surface, therefore we obtained the O_{top} as the pre-equilibrium surface. The PES and RDS of $Ru_5W_1O_X$ might be:

$$OH_{top} + O_{bri} \rightarrow \left(O_{top} + OH_{bri}\right) \rightarrow O_{top} + O_{bri} + H^+ + e^- \ (PES) \qquad (2)$$

$$O_{top} + O_{bri} + H_2O \rightarrow \left(OOH_{top} + OH_{bri}\right) \rightarrow OOH_{top} + O_{bri} + H^+ + e^- \ (RDS) \quad (3)$$

Since the intramolecular proton transfer was very fast in $Ru_5W_1O_x$, the overall RDS and PES were demonstrated as an electrochemical step.

For RuO_2, the analysis of the Tafel slope has been reported by several groups[8-10]. In our case, at the first Tafel region, the b = 54 mV dec^{-1}, $\beta = 1.09 \approx 1$, so the $\gamma = 1$, $n_{rds} = 0$. The Tafel slope of 54 mV dec^{-1} might be dominated by an electrochemical PES and a chemical RDS. Due to the strong H adsorption on Ru-O_{bri}-Ru, the pre-equilibrium surface at low overpotential might be partially protonated O_{top} + OH_{bri} surface[9]. The PES and RDS of RuO_2 might be:

$$O_{top} + OH_{bri} \rightarrow O_{top} + O_{bri} + H^+ + e^-\ (PES) \quad (4)$$

$$O_{top} + O_{bri} + H_2O \rightarrow OOH_{top} + OH_{bri}\ (RDS) \quad (5)$$

Since the OH_{bri} in RuO_2 was occupied by hydrogen, the addition of another water molecule required the deprotonation of the OH_{bri}.

Supplementary Note 3: Analysis of NAP-XPS data

To identify the surface oxygen species on the catalytic surface, we carried out NAP-XPS measurements on $Ru_5W_1O_x$ and reference RuO_2 under different water vapor pressure. The experiment procedure was similar to the report by Rao *et al.* on single-crystal RuO_2[11]. Before the measurements, the powder sample was tableted and exposed under 0.1 mbar O_2 at 250 ℃ for 30 min to remove the adsorbed water carbon contaminations. The O_2 heat-treated sample was regarded as the starting surface of the measurements (Supplementary Figure 27 and Supplementary Figure 28). Then, different amounts of water vapor were injected into the XPS chamber to conduct the measurements. We selected 735 eV incident X-ray energy to identify the surface species as in the previous report. Note that due to the good electric conductivity of Ru-based oxides, no obvious charging effect was observed during our measurements.

The data analysis was carried out in CasaXPS software. To deconvolution the measured O 1*s* XPS spectra, we adopt the following criteria:

- All O 1*s* XPS spectra used Tougaard-type background line;
- Four types of surface oxygen were taken into consideration: the lattice oxygen from oxides (O_{lat}), the protonated bridging oxygen (OH_{bri}), the adsorbed water or adsorbed hydroxyl (OH_{top}), and the gas phase water ($H_2O_{(g)}$);
- The O_{lat} and OH_{bri} peaks were fit by GL(10) line shape, the OH_{top} and $H_2O_{(g)}$ peaks were fit by GL(25) line shape;
- The binding energy (BE) of O_{lat} and OH_{bri} was restricted by: BE(OH_{bri}) = BE(O_{lat}) + 1.2 eV for $Ru_5W_1O_x$, BE(OH_{bri}) = BE(O_{lat}) + 1.5 eV for RuO_2.

The detailed peak profiles were listed in Supplementary Table 3 and Supplementary Table 4.

Due to the strong interaction between water and RuO_2 surface[12], the water molecules tend to dissociatively adsorb onto the RuO_2 surface by transferring protons onto the adjacent O_{bri}. Therefore, once the water vapor was injected, the OH_{top} peak and OH_{bri}

peak of RuO_2 increased, leading to high coverage of OH*. When the water vapor pressure increased from the ultra-high vacuum (UHV) to 1 mbar, the peak area ratio of OH_{bri}:O_{lat} increased accordingly from 2.7 to 3.9 (Supplementary Figure 29d). When returned to the UHV, the gas-phase water and adsorbed water was removed, but the OH_{bri} peak remained, indicating the strong H^+ binding on the Ru-O_{bri}-Ru sites. To remove these adsorbed H^+, extra energy input was needed. We also examined the Ru 3*d* XPS spectra under the same conditions (Supplementary Figure 30). We noticed that the $3d^{5/2}$ peak of RuO_2 moved to lower BE as the pressure increased. We attributed this to the protonation of surface Ru-O_{bri}-Ru sites, which will lead to a decrease in the average chemical state of surface Ru atoms.

On the $Ru_5W_1O_x$, the situation was different from that on pure RuO_2. The low H^+ adsorption energy on W-O_{bri}-Ru led to lower OH_{bri} content than RuO_2 (Supplementary Figure 29c). Instead of the valence change of surface Ru atoms, we observed the valence change of W atoms along with the pressure change (Supplementary Figure 31). This verified that water molecules dissociatively adsorbed on the catalytic surface by transferring protons to the W-O_{bri}-Ru (or W-O_{bri}-W) sites. The adsorption of water led to an increase in W^{5+} content (Supplementary Table 5). Different from the pure RuO_2, the protonation/deprotonation on W-O_{bri}-Ru was highly reversible. When reducing the water vapor pressure, the OH_{bri} and W^{5+} content decreased accordingly. We believe this mild proton adsorption will contribute to the deprotonation of oxo-intermediates during OER, thus the accelerated overall OER kinetics.

Supplementary Note 4: In-situ electrochemical XPS

For the in-situ electrochemical XPS measurements, we developed a two-electrode static electrochemical cell adapt to the NAP-XPS chamber of the BL02B01 beamline of SSRF (Supplementary Figure 32). The design of the electrochemical cell is similar to the report by Falling *et al.*[13], which used a membrane electrode assembly (MEA) as the working electrode. The fabrication of the MEA followed the conventional catalyst-coated membrane (CCM) methods. At the anode side, the catalyst powder was mixed with Nafion ionomers and sprayed onto the Nafion 117 membrane. The membrane was hot-pressed and then boiled in 0.5M H_2SO_4 and DI water. A gold (Au) coated Ti lid was used as the anode current collector, the gold film can be used to calibrate the binding energy if there was any charging effect. A Pt electrode was used as the counter and reference electrode, which was grounded to the electron energy analyzer so that the potential of the working electrode can be controlled by a potentiostat. The pressure of the XPS chamber was balanced at 0.25 mbar by injecting some water vapor. The incident energy of the X-ray was set to 735 eV. Before the measurements, three CV scans between 0.1 V to 1.7 V were conducted to remove the contaminations on the catalyst surface. During the in-situ measurements, we moved the beam back and forth between the sample and Au-coated lid at each applied potential. We noted that the

potential of the working electrode can be precisely controlled (Supplementary Figure 33). For example, when applying 1.6 V, the BE of Au $4f^{7/2}$ peak would increase by 1.6 eV to 85.6 eV, and so did the BE of measured catalyst samples. We, therefore, aligned the BE of all samples according to the Au $4f^{7/2}$ peak at 84.0 eV in the following discussions.

To investigate the property of surface O_{bri} sites, we focus on the change of O 1*s* spectra during in situ measurements. Different from the ex-situ NAP-XPS measurements, under in situ conditions, the contribution of the adsorbed water/liquid phase water (denoted as $H_2O_{(l)}$) at the surface became dominant[14]. We, therefore, deconvolved the O 1*s* signals based on the following criteria:

- Tougaard type background line;
- Four types of surface oxygen were taken into consideration: the lattice oxygen from oxides (O_{lat}), the protonated bridging oxygen (OH_{bri}), water/liquid phase water ($H_2O_{(l)}$), and the gas phase water ($H_2O_{(g)}$);
- The O_{lat} and OH_{bri} peaks were fit by GL(10) line shape, the OH_{top} and $H_2O_{(g)}$ peaks were fit by GL(25) line shape;
- The BE of O_{lat} and OH_{bri} were restricted by $BE(OH_{bri}) = BE(O_{lat}) + 1.5$ eV (same with the ex-situ results);
- The BE of $H_2O_{(l)}$ and $H_2O_{(g)}$ were restricted by $BE(H_2O_{(l)}) = BE(H_2O_{(g)}) - 2.2$ eV (obtained from ref.[15]);

The detailed peak profiles were listed in Supplementary Table 6 and Supplementary Table 7.

Based on the above criterion, we were able to distinguish the change of OH_{bri} along with the electric potential on the $Ru_5W_1O_x$ catalyst. As the potential increased, the OH_{bri} peak diminished accordingly, indicating the deprotonation of bridging oxygen. At OER potential (1.6 V), the O_{bri} sites were fully deprotonated. When reducing the potential back to 0.8 V, the O_{bri} sites were again protonated. This proved the reversible protonation/deprotonation features of W-O_{bri}-Ru sites, as predicted by DFT calculations.

For RuO_2, due to the strong hydroxylation of the catalyst surface, we did not observe such deprotonation processes (Supplementary Figure 35). We attribute the observed peak (at *ca.* 532 eV) to the hydroxyl adsorbed on the coordinate coordinatively unsaturated Ru sites (Ru_{CUS}).

Supplementary Note 5: The modeling of electrolyte/catalyst interface

To establish a reasonable model for acid conditions, we used a water box including Cl^- anions to represent the counter ions. The distance between the Cl^- ion and the RuO_2 surface is about 2 Å. Note that there is a large number of possible proton transfer pathways, and the process is accompanied by the change of solvation energy and configurational entropy. It is beyond the scope of the current study to investigate all possibilities. Our barriers, therefore, represent upper bounds to the minimum energy

barriers. To investigate the proton transfer barrier, we considered different water molecules as the proton carrier (H_3O^+) and compared proton transfers to bulk water in systems with and without acid ions. Due to finite cell size effects, the dipole created by the charge transfer forces the H^+ to be attracted to the surface, where it adsorbs. As this is not the final state expected under oxidizing conditions, we stabilize the H^+ by the presence of an anion in the water. For this reason, we reported only barriers calculated in the presence of a Cl^- ion.

Using the direct relationship between the optimized electrode surface and solvent molecules, we found that there is obvious hydrogen bonding (the distance between O in H_2O and H in OH_{bri} is 1.03 Å and 1.57 Å on the surface of RuO_2 and W-doped RuO_2, respectively) between the first layer water molecules and OH_{bri}, as shown in Supplementary Figure 43b and e. Such hydrogen bonding makes it easier for the OH_{bri} to detach from the surface and form protons (H_3O^+) in electrolytes. The proton transfer energy barrier calculated with the climbing image nudged elastic band (CI-NEB) method[16] has been shown in Fig. 5b. We set up 9 images between the initial and final state, and the transition state (TS) on both RuO_2 and W-doped RuO_2 appear at the first image. In other words, the adsorbed H on the O_{bri} site can be desorbed rapidly within 0.1–0.2 eV, which may be related to the enthalpy barrier and the transfer of proton into the outer Helmholtz layer[17].

In particular, W-doped RuO_2 presents moderate $\triangle G_H$, showing a lower proton transfer energy barrier (0.11 eV) than RuO_2 (0.23 eV). Surface charge distribution calculation shows that the H atom on the W-doped RuO_2 surface (+0.144 |e|) presents a more positive charge than the H atom on the RuO_2 surface (+0.116 |e|), which can support the conclusion that H atoms adsorbed on Ru-O_{bri}-W are more likely to be deprotonated than those adsorbed on Ru-O-Ru.

In addition, the FS state shows lower energy than IS state. The reason is that H_3O^+ is an energy-favorable state. There is a balance between H_3O^+ and $H_5O_2^+$, where $H_5O_2^+$ shows higher energy than H_3O^+ (ref.[18]).

Supplementary Figures

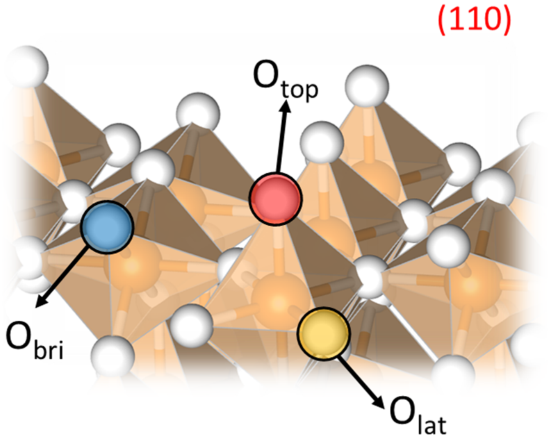

Supplementary Figure 1 | Schematic of different oxygen sites on the RuO_2 (110) surface. Three different oxygen sites can be distinguished on the (110) surface of RuO_2: the on-top oxygen (O_{top}) which bonded with one Ru atom, the bridging oxygen (O_{bri}) which bonded with two Ru atoms, and the lattice oxygen (O_{lat}) which bonded with three atoms.

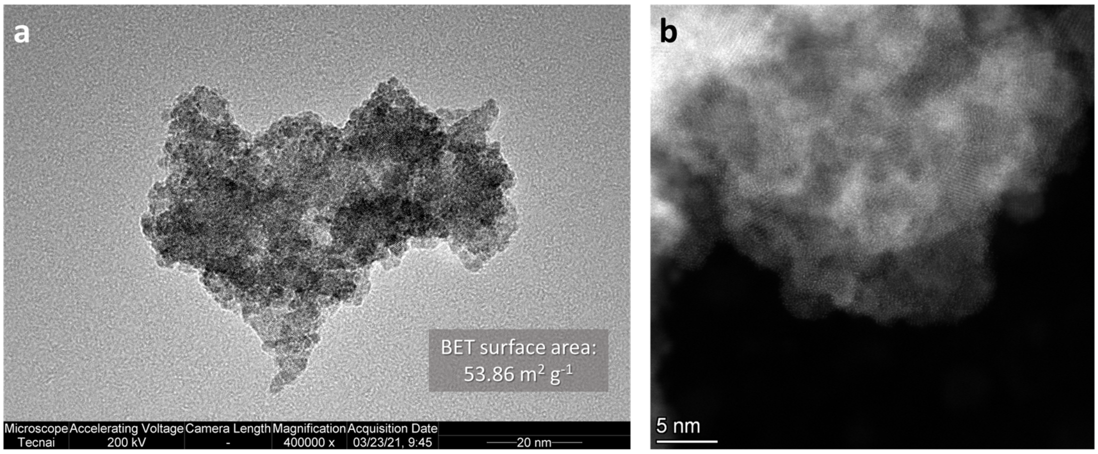

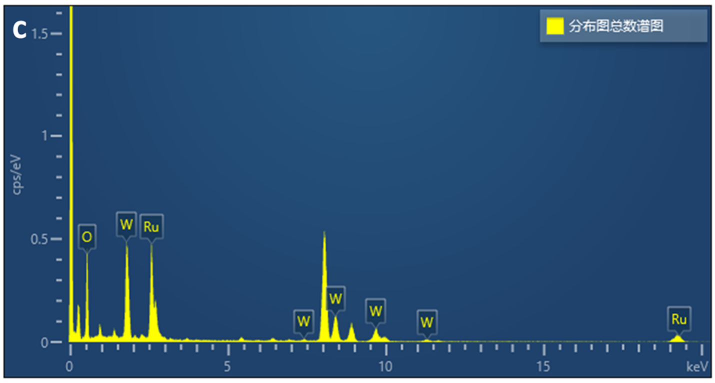

d

Element	Atomic %
Ru	24.51
W	9.17
O	66.31

Supplementary Figure 2 | The morphology and composition of $Ru_5W_1O_x$ before OER. **(a)** The TEM image. Scale bar: 20 nm. **(b)** The STEM-HAADF image. Scale bar: 5 nm. **(c-d)** The EDX spectrum and atomic ratio before OER.

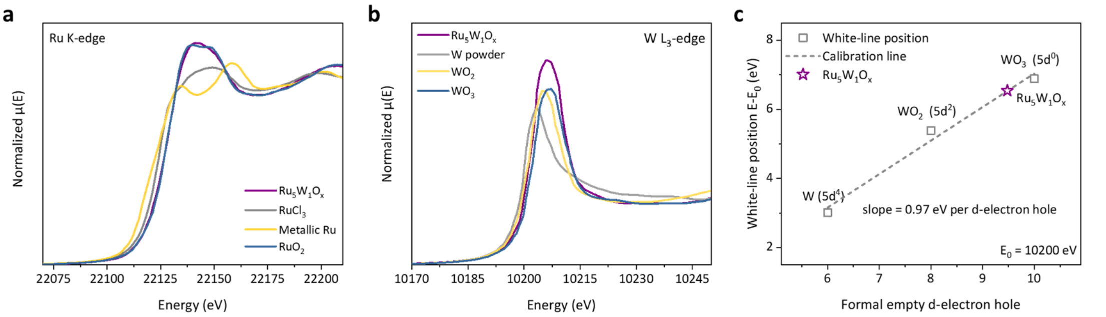

Supplementary Figure 3 │ The XANES spectra of $Ru_5W_1O_x$ and reference materials. **(a)** Ru K-edge XANES spectra. The Ru in $Ru_5W_1O_x$ showed a chemical state of +4, almost the same as with RuO_2 reference. **(b)** The W L_3-edge XANES spectra. The white-line peak of $Ru_5W_1O_x$ was located between the WO_2 and WO_3 samples. **(c)** White-line position of $Ru_5W_1O_x$ (Pentastar) as a function of the formal *d*-electron hole numbers. Formal empty *d*-electron hole values were calculated based on the white-line shift and the increase of 0.97 eV per *d*-electron hole calibrated from W powder ($5d^4$), WO_2 ($5d^2$) and WO_3 ($5d^0$) reference samples. $Ru_5W_1O_x$ demonstrated a chemical state between +5 and +6. We attributed the +5 part to the proton-occupied W-OH_{bri}-Ru sites.

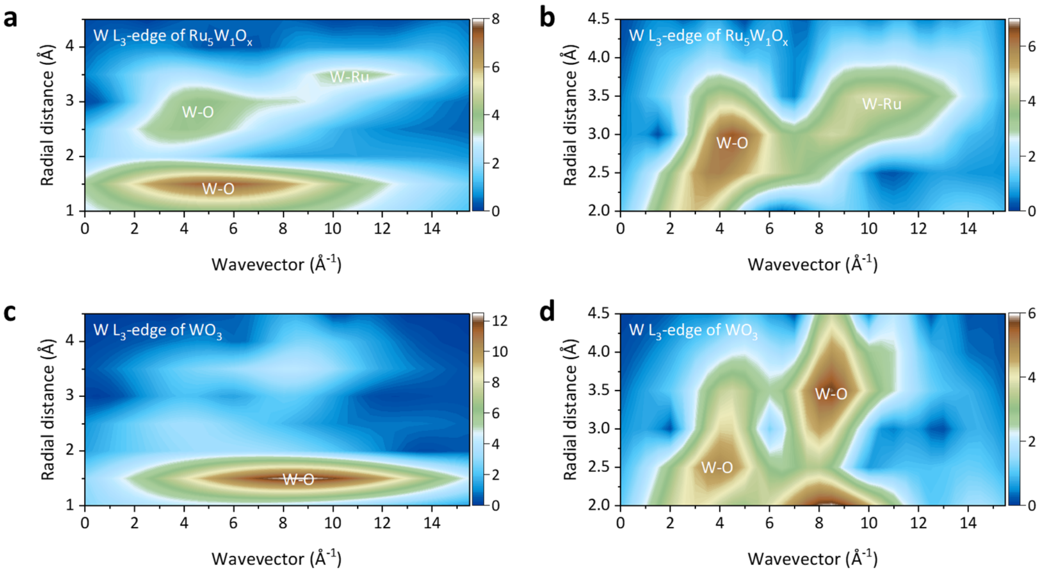

Supplementary Figure 4 | The wavelet transformation (WT) of k^3-weight W L_3-edge EXAFS data. We used Morlet wavelets as the mother wavelets. **(a-b)** WT-EXAFS of $Ru_5W_1O_x$. **(a)** is the overview graph. **(b)** is the zoom of the second coordination shell. **(c-d)** WT-EXAFS of WO_3 reference. **(c)** is the overview graph. **(d)** is the zoom of the second coordination shell. Morlet function parameters: η = 5.3, σ = 2.3 for the overview graph, η = 6.3, σ = 1.2 for the zoom graph. $Ru_5W_1O_x$ showed different first shell and second shell coordination from WO_3. The wavelet maximum at **R** ≈ 3.5 Å, **k** ≈ 11 $Å^{-1}$ can be attributed to the W-Ru scattering.

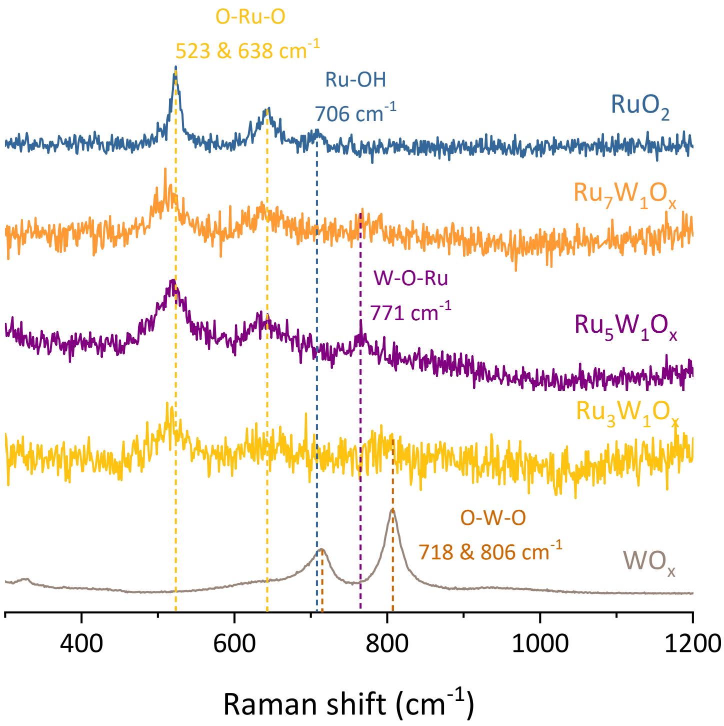

Supplementary Figure 5 | The Raman spectra of different materials. In RuO_2, the peak at 523 cm^{-1} (E_g) and 638 cm^{-1} (A_{1g}) can be assigned to the O-Ru-O vibration[19], and the peak at 706 cm^{-1} (B_{2g}) may come from Ru-OH vibration[20]. In WO_x, the peaks at 718 cm^{-1} and 806 cm^{-1} were contributed by the O-W-O stretching modes[21]. In $Ru_5W_1O_x$, the E_g and A_{1g} peak remained, but the B_{2g} peak became less prominent. Instead, a peak located at 771 cm^{-1} appeared. Compared to the vibration modes in RuO_2 and WO_x, we attribute this peak to the W-O-Ru vibration.

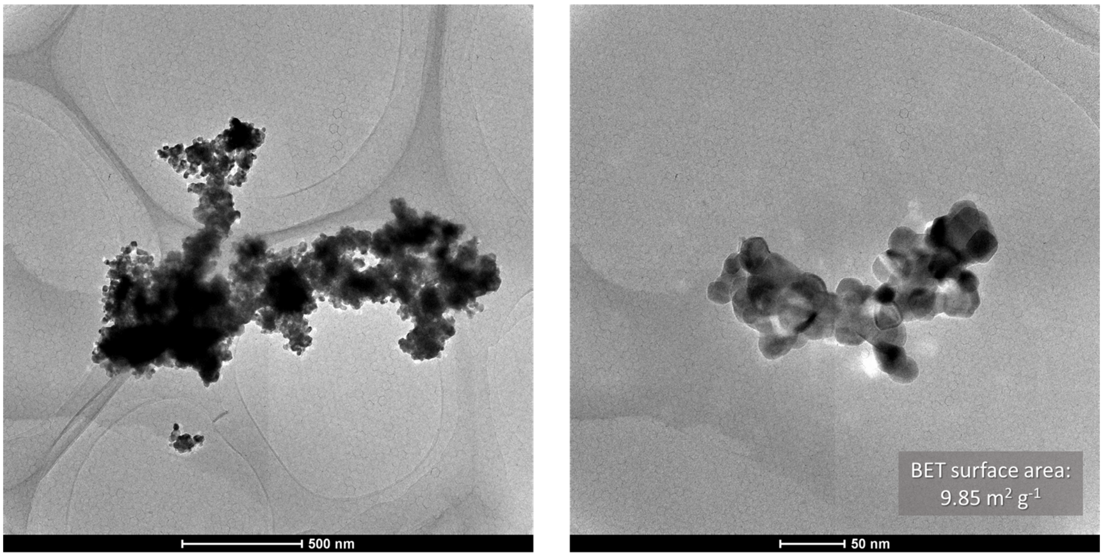

Supplementary Figure 6 | TEM images of commercial RuO_2 nanoparticles. ~20 nm nanoparticles, with a BET surface area of 9.85 m^2 g^{-1}.

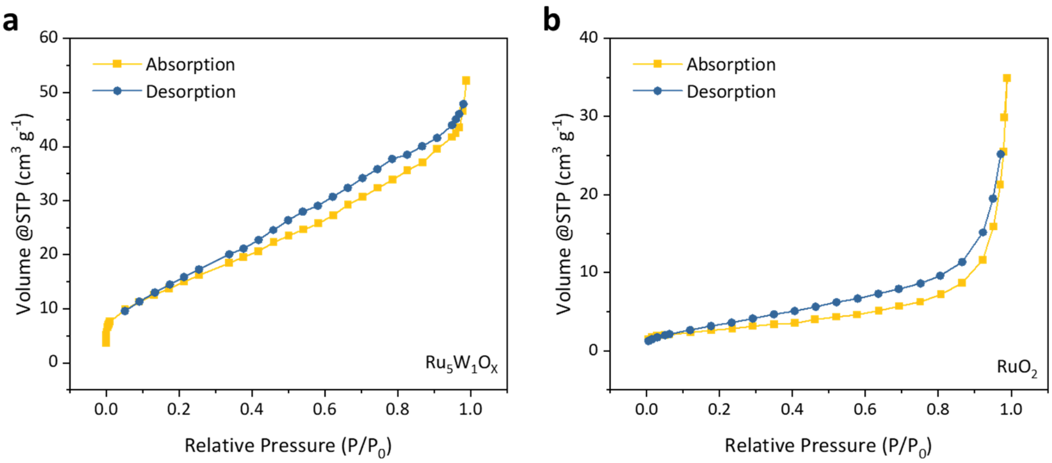

Supplementary Figure 7 | The BET isotherm plots of different catalysts. (a) $Ru_5W_1O_x$. **(b)** Commercial RuO_2.

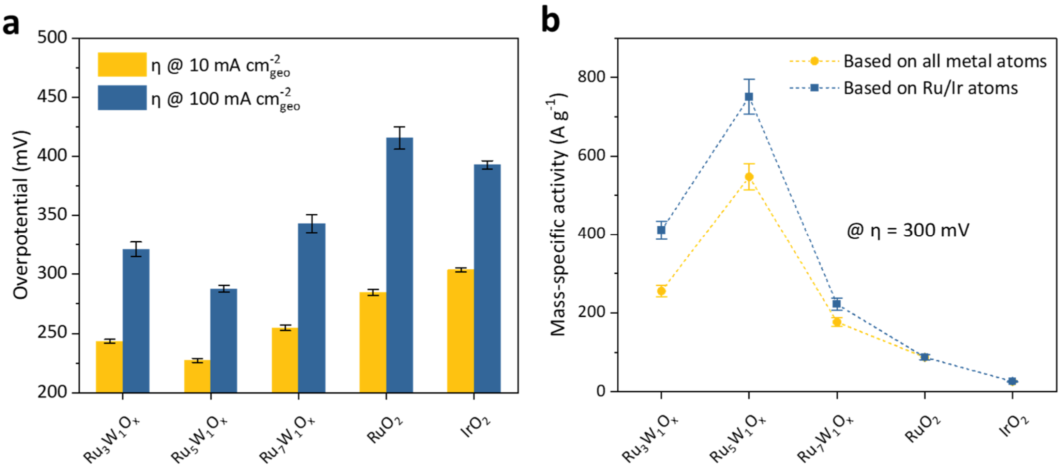

Supplementary Figure 8 | OER performance evaluation of different catalysts. (a) The overpotential to reach 10 mA cm^{-2} and 100 mA cm^{-2} OER current. (b) The mass-specific activity was calculated at η = 300 mV according to total Ru loadings or metal loadings. The error bars were standard deviations of averaging three independent measurements.

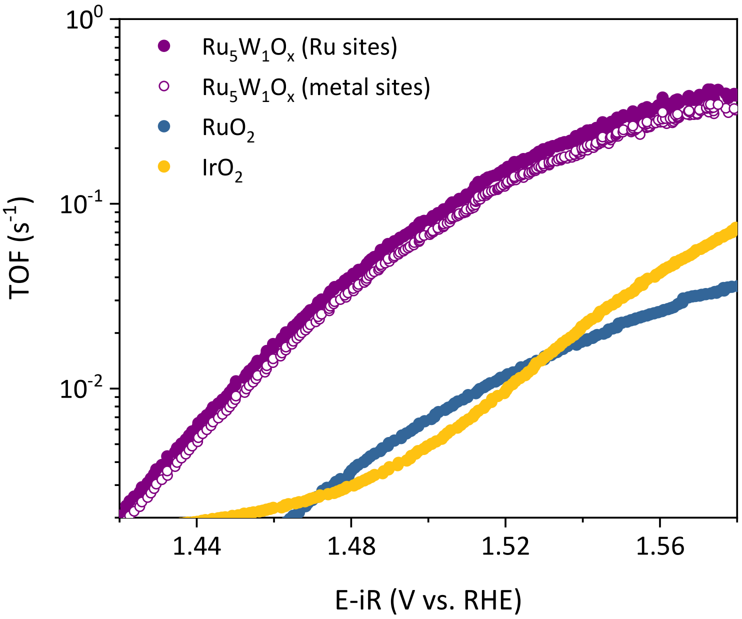

Supplementary Figure 9 | The turnover frequencies (TOF) of different catalysts. The TOF values are calculated by assuming all metal atoms loaded are active sites. The turnover frequency (TOF) of $Ru_5W_1O_x$ reached 0.163 ± 0.010 s^{-1} (at η = 300 mV), which is higher than the pristine RuO_2 (0.007 ± 0.002 s^{-1}) and nano-IrO_2 (0.012 ± 0.001 s^{-1}). The TOF values of $Ru_5W_1O_x$ calculated by assuming all Ru atoms as active sites are also provided in the figure, which is 0.195 ± 0.011 s^{-1} at η = 300 mV. The standard deviations are calculated by averaging three independent measurements.

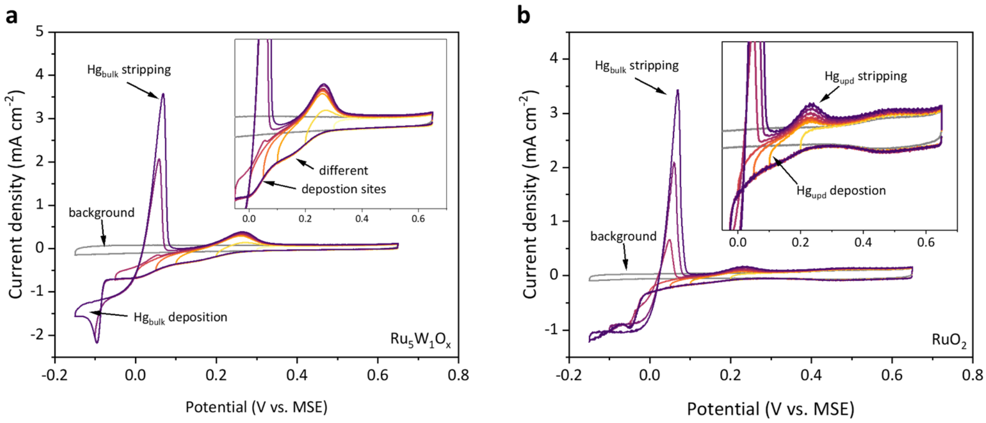

Supplementary Figure 10 | Determining the ECSA using mercury underpotential deposition. The cyclic voltammetry of **(a)** $Ru_5W_1O_x$ and **(b)** RuO_2 at 50 mV s^{-1} scan rate. The experiment was conducted in 0.1 M $HClO_4$ containing 1 mM $Hg(NO_3)_2$. The current difference of the cathodic scans between Hg-containing solution and blank background was integrated to calculate the amount of Hg_{upd}. A coulombic charge of 138.6 μC cm^{-2} was used as a factor to obtain the ECSA values.

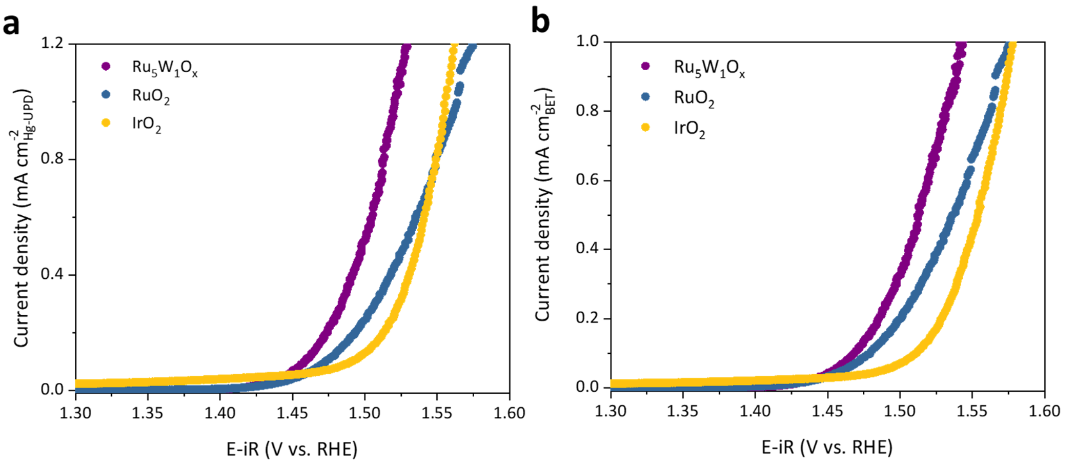

Supplementary Figure 11 | Evaluation of specific OER activity. (a) Normalized by ECSA (determined by mercury underpotential deposition). **(b)** Normalized by BET surface area of the catalyst powder.

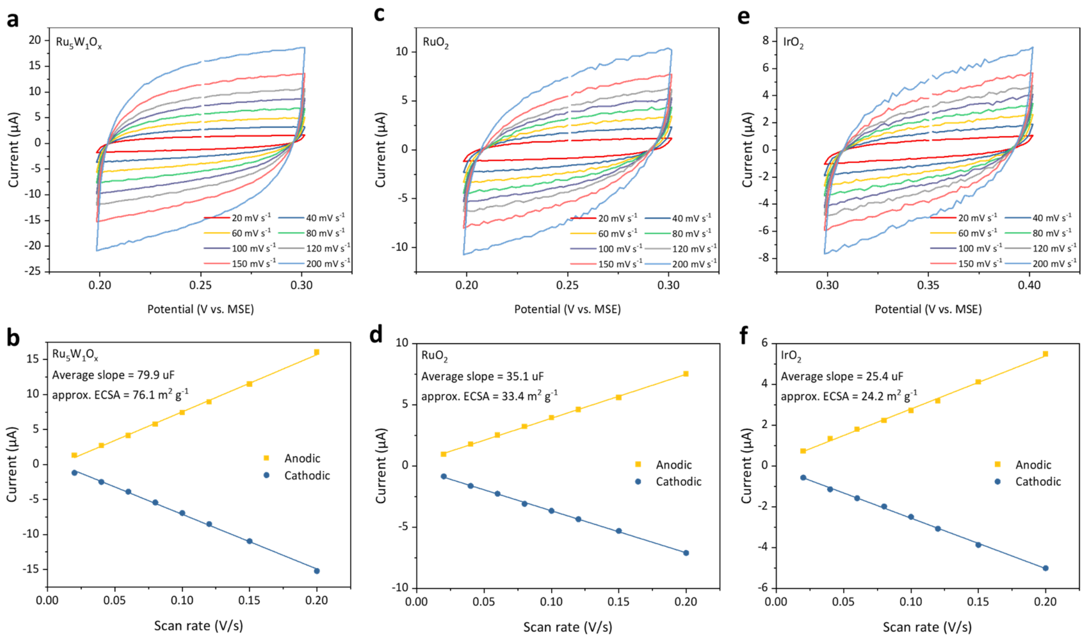

Supplementary Figure 12 | Determination of C_{dl} by Cyclic voltammetry. (a-b) $Ru_5W_1O_x$. **(c-d)** RuO_2. **(e-f)** IrO_2. Cyclic voltammetry scanned between 0.20 to 0.30 V *vs.* MSE. The cathodic and anodic charging currents measured at 0.25 V *vs.* MSE were plotted as a function of scan rate. A general specific capacitance (C_s) of 35 µF cm^{-2} was used to calculate ECSA.

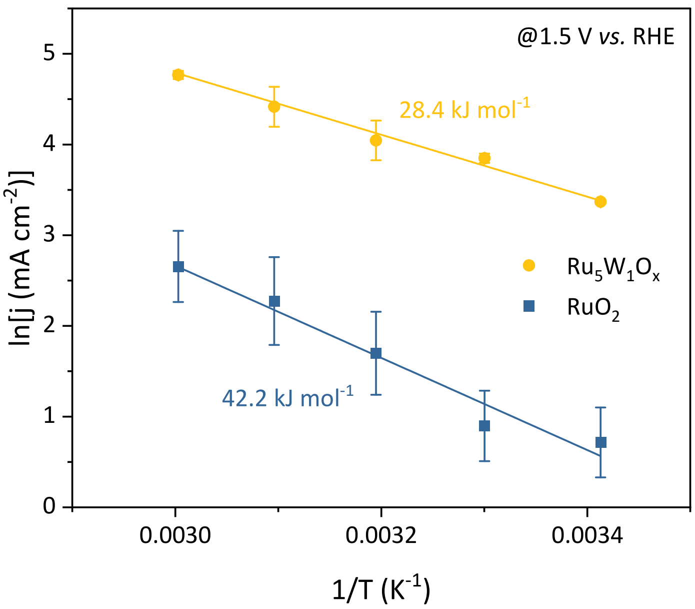

Supplementary Figure 13 | The apparent activation energy of $Ru_5W_1O_x$ and RuO_2. Calculated according to the current density at 1.50 V *vs.* RHE. The error bars were standard deviations of averaging three independent measurements.

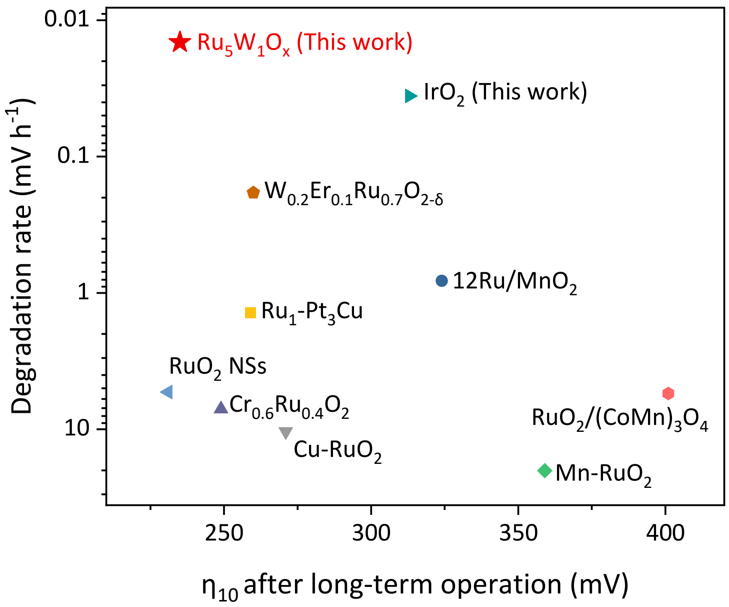

Supplementary Figure 14 | Performance of iridium-free OER catalysts in acidic electrolytes. The corresponding references are summarized in Supplementary Table 8.

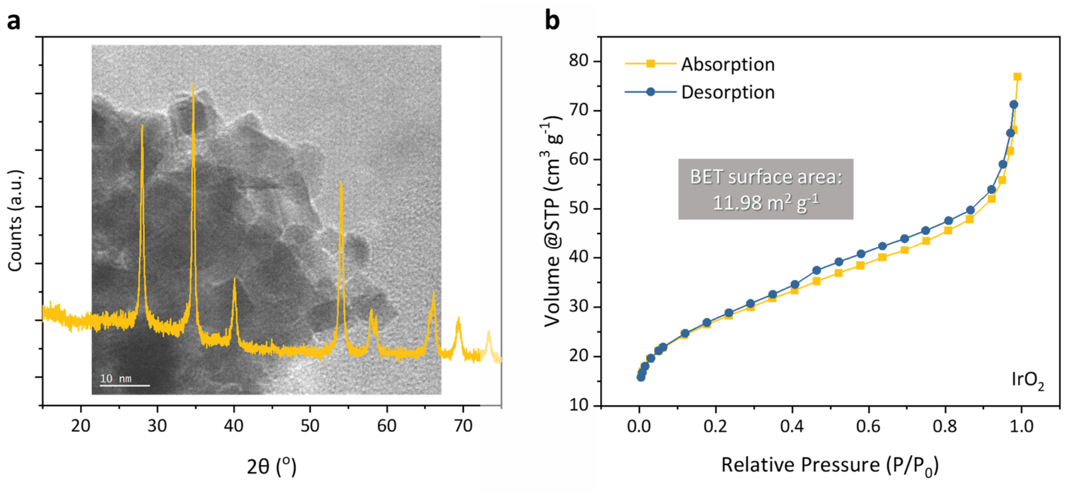

Supplementary Figure 15 | The characterizations of the commercial IrO_2 catalyst. **(a)** The TEM image and XRD pattern. **(b)** The BET isotherm plot.

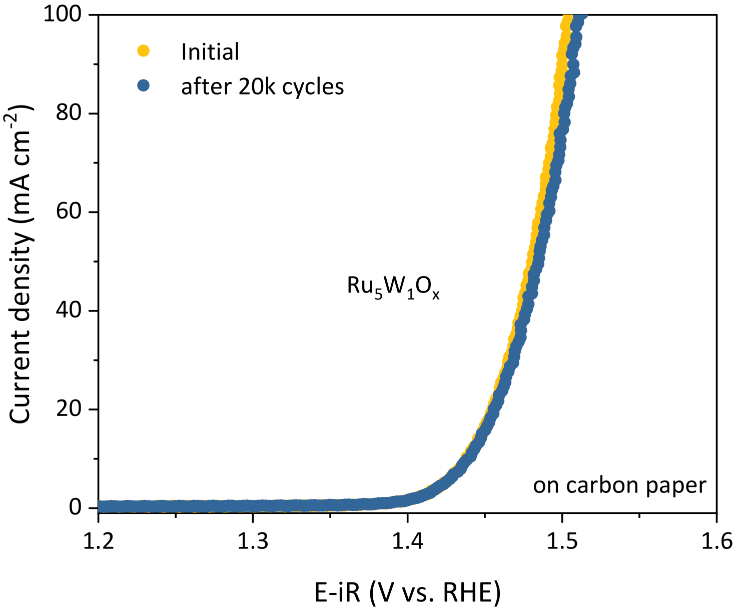

Supplementary Figure 16 | The LSV curves of $Ru_5W_1O_x$ before and after 20,000 CV cycles. Potential ranges between 0.95 to 1.55 V *vs.* RHE. Scan rate: 5 mV s^{-1}. Measured on carbon paper.

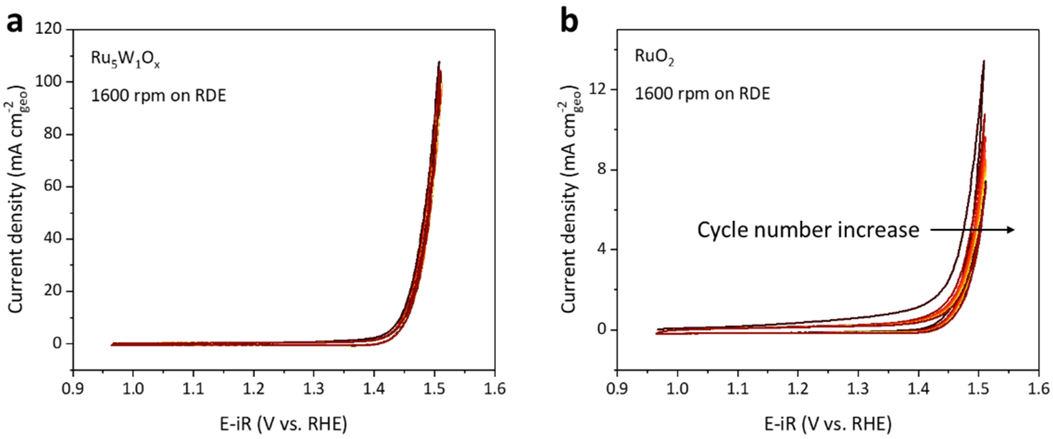

Supplementary Figure 17 | 10 CV cycles of different catalysts before LSV test. (a) $Ru_5W_1O_x$. **(b)** RuO_2. Scan rate: 50 mV s^{-1}.

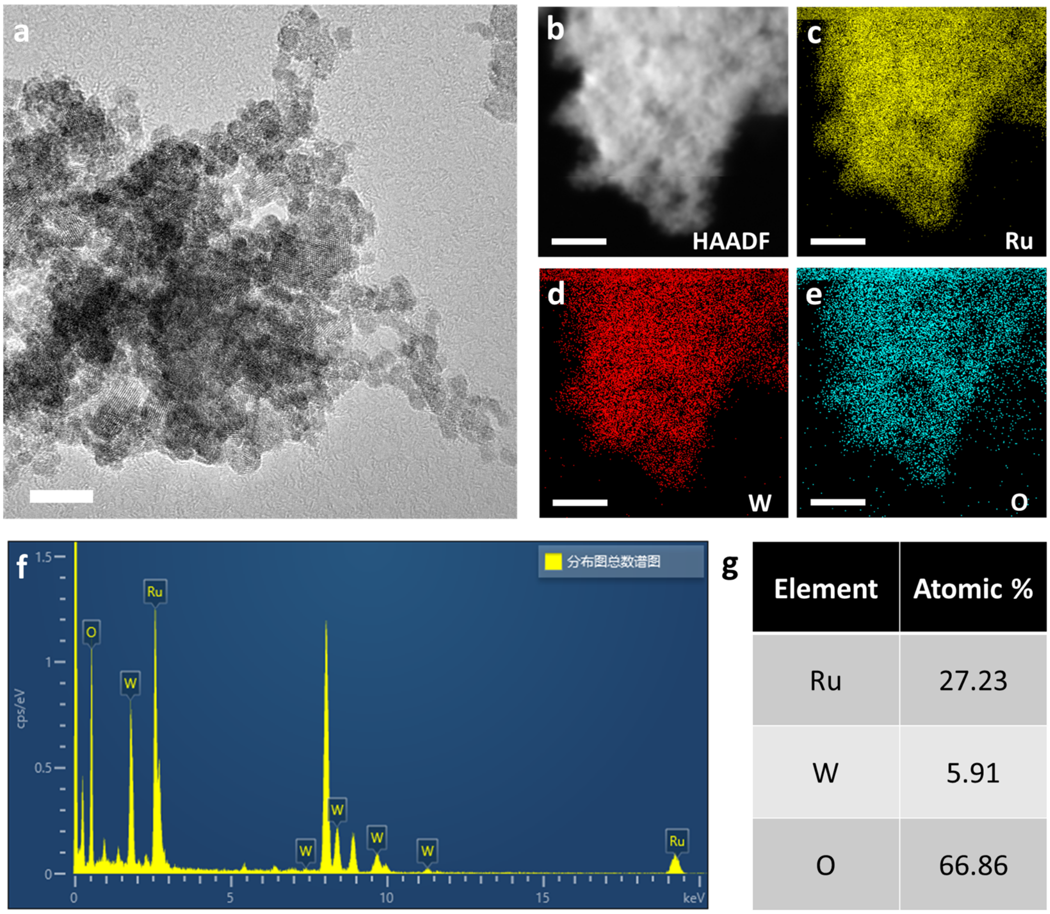

Element	Atomic %
Ru	27.23
W	5.91
O	66.86

Supplementary Figure 18 | The morphology and composition of $Ru_5W_1O_x$ after 12 h chronopotentiometry test at 10 mA cm^{-2}. (a) The TEM image. Scale bar: 10 nm. **(b-e)** The STEM-HAADF image and corresponding element mapping. **(f-g)** The EDX spectrum and atomic ratio after OER.

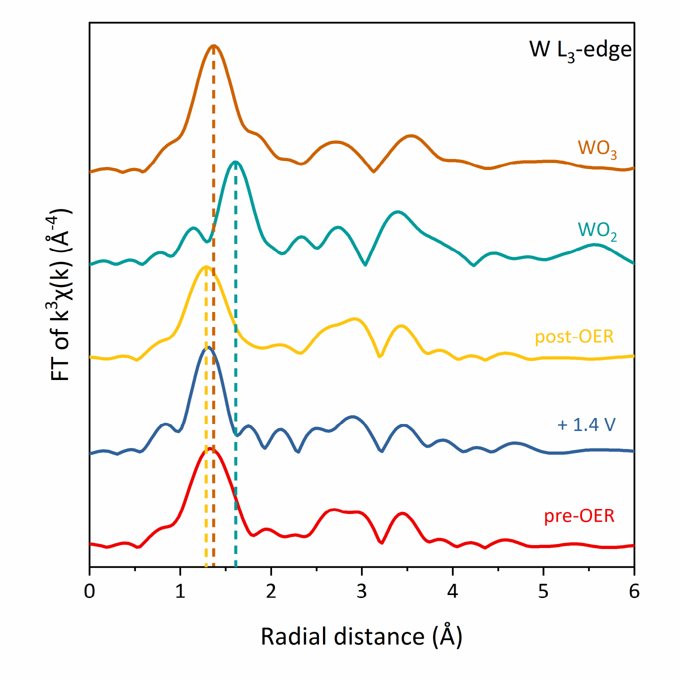

Supplementary Figure 19 | The *in situ* W L_3-edge EXAFS spectra of $Ru_5W_1O_x$. When applied the OER potential (1.4 V *vs.* RHE), the local structure did not change significantly. Comparing the spectra before and after OER, we noticed a slight decrease in W-O distance. We attributed this change to the chemical valence change of some W sites in the catalyst, which coincided with the results of AP-XPS, and the W-O structure in the RuO_2 matrix was kept stable under OER conditions.

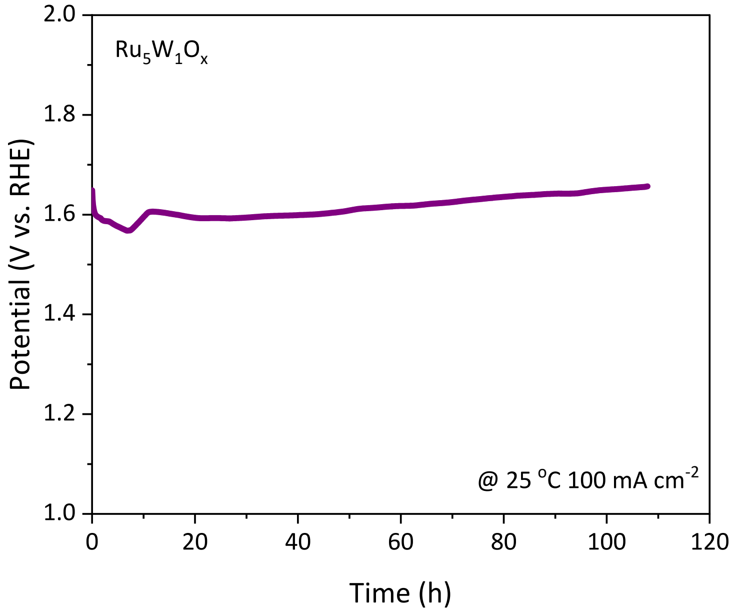

Supplementary Figure 20 | Chronopotentiometry of $Ru_5W_1O_x$ at 100 mA cm^{-2}. Using carbon paper as the substrate, without iR compensation. Since the carbon paper

will suffer from more severe corrosion under high current density in H_2SO_4, we used 1 M $HClO_4$ as the electrolyte in the high current density test[22,23].

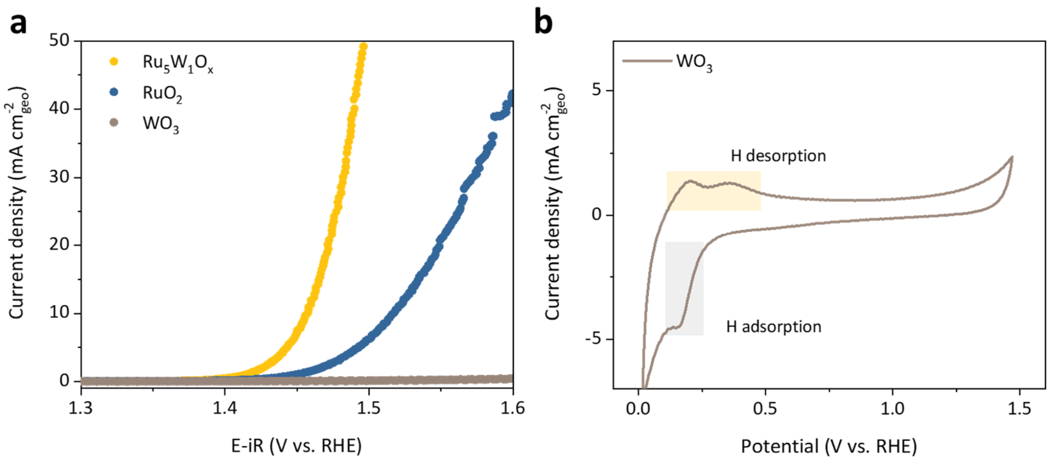

Supplementary Figure 21 | The electrochemistry of homemade WO_3. (a) The OER polarization curves of different catalysts. Scan rate: 5 mV s^{-1}. The WO_3 showed almost no OER reactivity. We postulated that the W surrounded by Ru may contribute negligibly to the OER. **(b)** The CV profile of WO_3. Scan rate: 200 mV s^{-1}. The shaded area showed the hydrogen adsorption and desorption of WO_3.

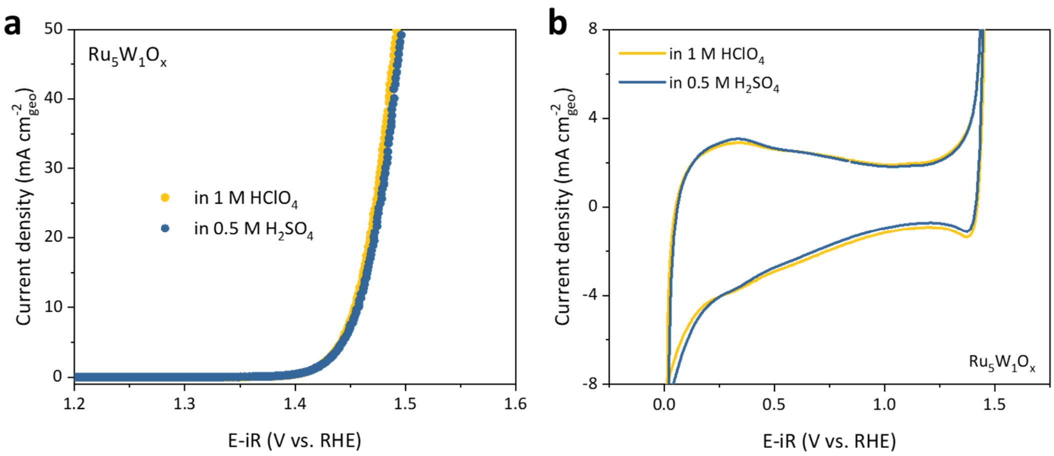

Supplementary Figure 22 | The electrochemical behavior of $Ru_5W_1O_x$ in different electrolytes. (a) LSV curves. **(b)** CV profiles.

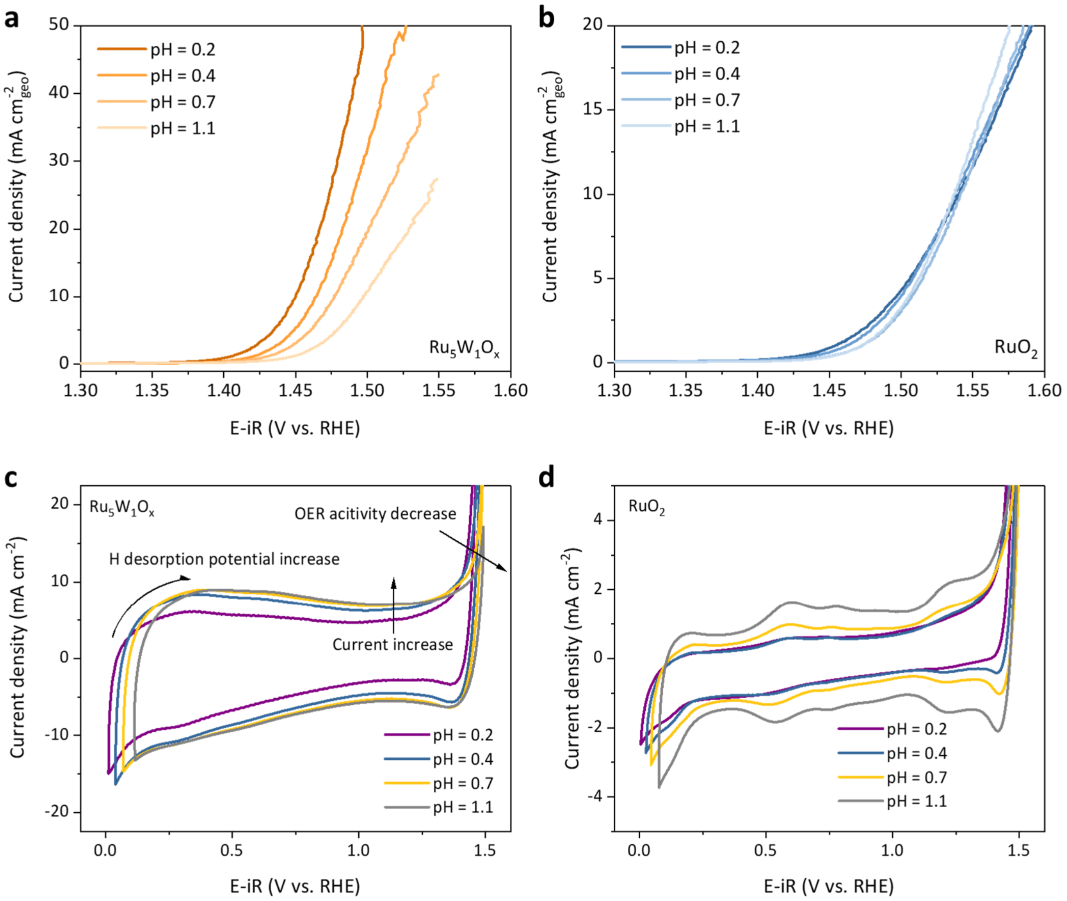

Supplementary Figure 23 | pH dependence of OER activity. (a-b) The LSV curves of $Ru_5W_1O_x$ and RuO_2 at different pH. Scan rate: 5 mV s^{-1}. **(c-d)** The CV curves of $Ru_5W_1O_x$ and RuO_2 at different pH. Scan rate: 200 mV s^{-1}.

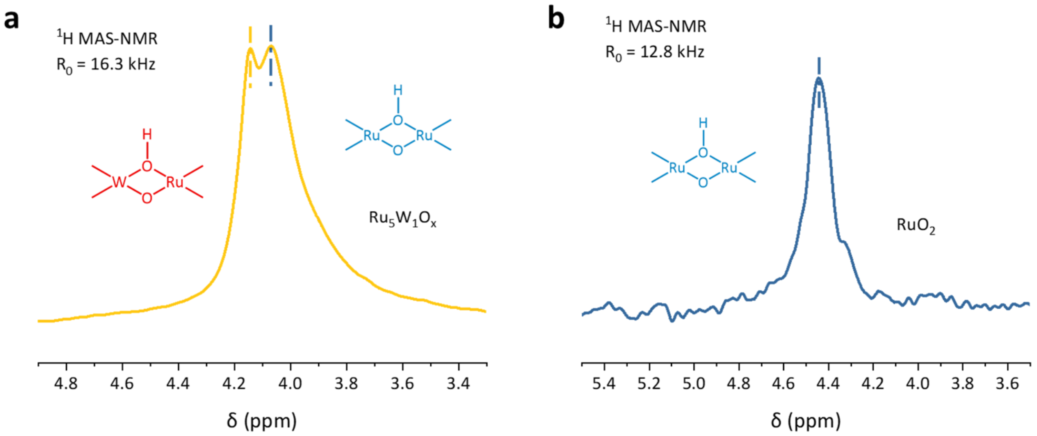

Supplementary Figure 24 | The ^{1}H magic-angle spin nuclear magnetic resonance (MAS-NMR) spectra of different catalysts. (a) $Ru_5W_1O_x$. **(b)** RuO_2. R_0 refers to the MAS rotation speed.

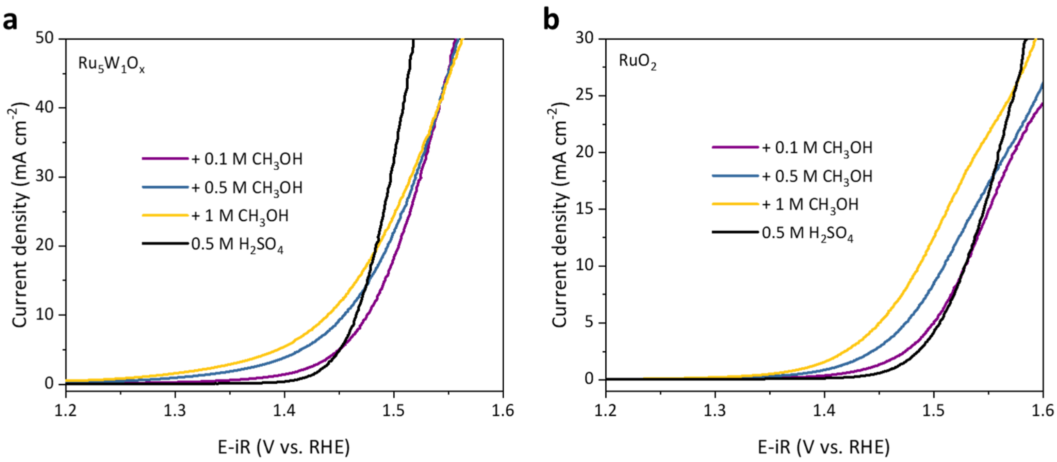

Supplementary Figure 25 | The MOR performance of different catalysts. (a) $Ru_5W_1O_x$. **(b)** RuO_2. Scan rate: 5 mV s^{-1}. MOR and OER are competition reactions. On the OH* covered surface, MOR is preferred. After surface OH* is deprotonated, the OER is preferred. In $Ru_5W_1O_x$, the OH_{bri} deprotonated at low overpotential, thus favoring OER. In RuO_2, the deprotonation is difficult, thus favoring MOR.

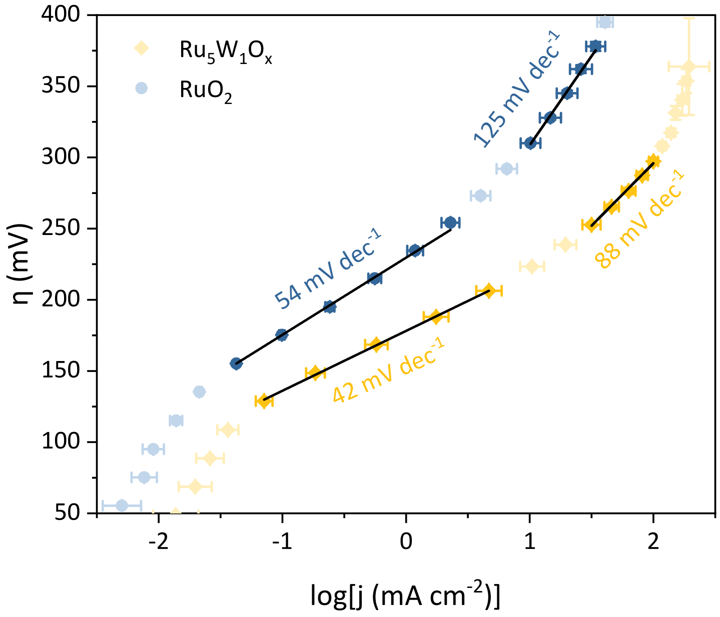

Supplementary Figure 26 | The Tafel plots of different catalysts. The error bars were standard deviations of averaging three independent measurements. The detailed discussion of Tafel's behavior refers to Supplementary Note 1.

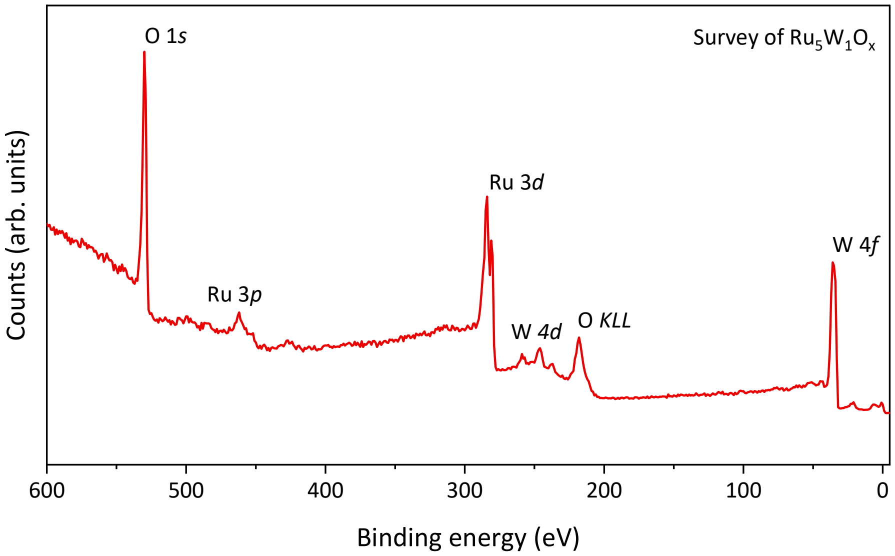

Supplementary Figure 27 | The XPS survey of $Ru_5W_1O_x$ under UHV. The sample was first cleaned under 5 mbar O_2 at 250 ºC to remove the carbon contaminations and adsorbed water.

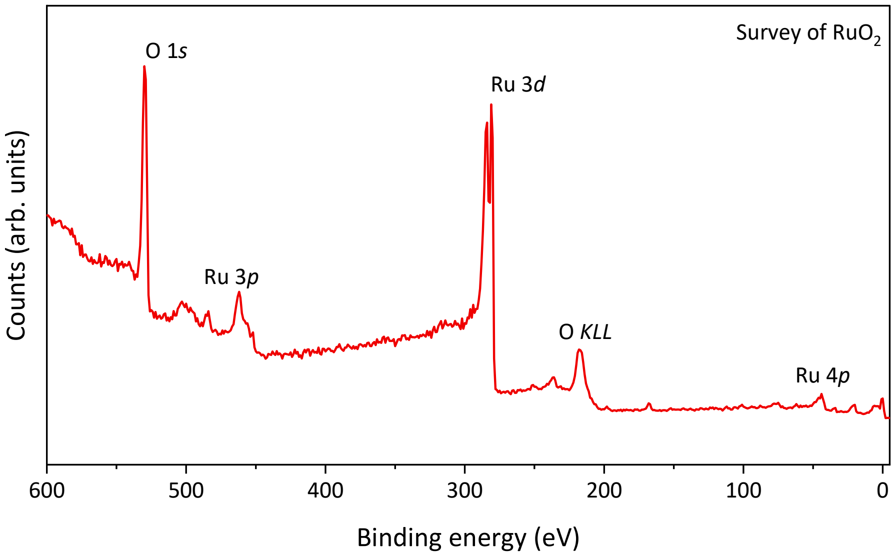

Supplementary Figure 28 | The XPS survey of RuO_2 under UHV. The sample was first cleaned under 5 mbar O_2 at 250 ºC to remove the carbon contaminations and adsorbed water.

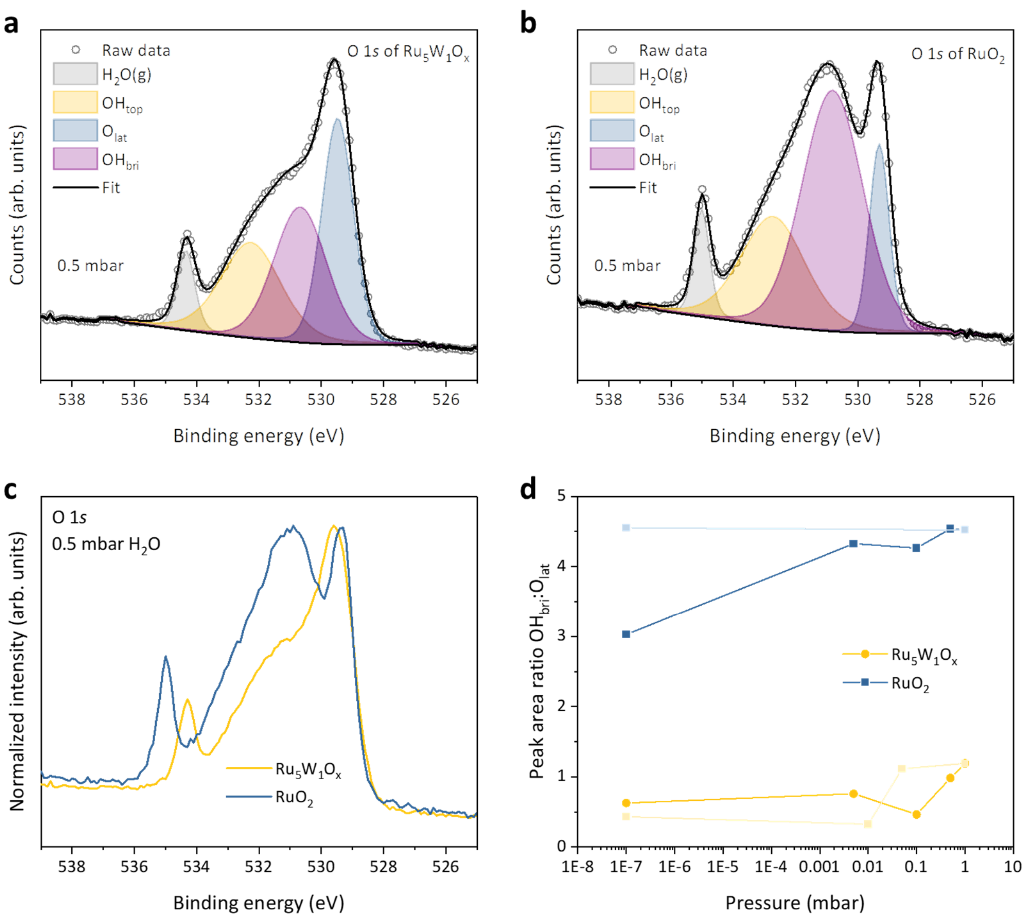

Supplementary Figure 29 | Analysis of O 1*s* XPS spectra. (a-b) The typical deconvolution of O 1*s* XPS spectra of $Ru_5W_1O_x$ and RuO_2. **(c)** Direct comparison between two catalysts. The binding energy difference of the gas-phase water peak may be caused by the different work functions of the samples. **(d)** The OH_{bri}:O_{lat} peak ratio as a function of water vapor pressure. The UHV condition is regarded as 1×10^{-7} mbar. The summary of peak deconvolution parameters refers to and Supplementary Table 4.

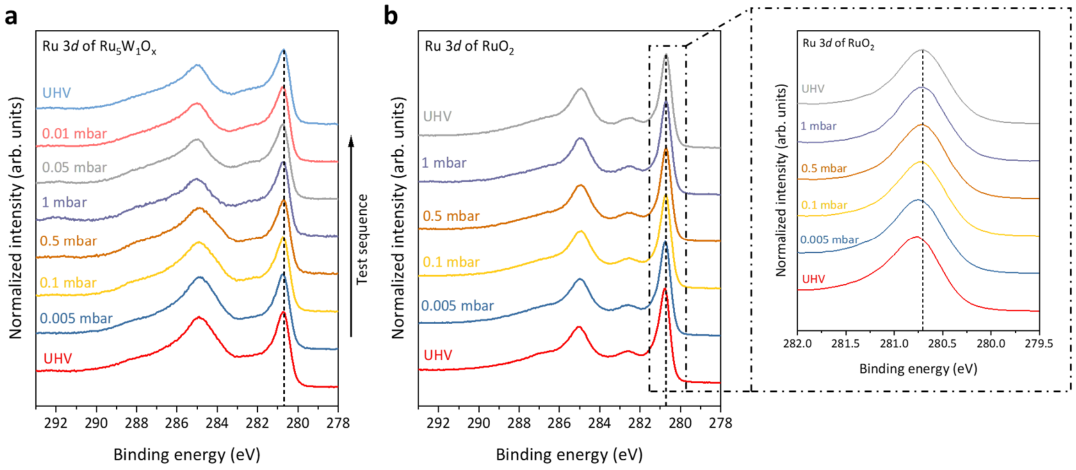

Supplementary Figure 30 | Analysis of Ru 3*d* XPS spectra. (a) The Ru 3*d* spectra of $Ru_5W_1O_x$ at different conditions. **(b)** The Ru 3*d* spectra of RuO_2 at different conditions.

The zoom area showed that the binding energy of the Ru $3d^{5/2}$ peak decreased slightly along with the pressure change, which could be due to the increase of OH_{bri} species slightly reducing the valence of surface Ru atoms.

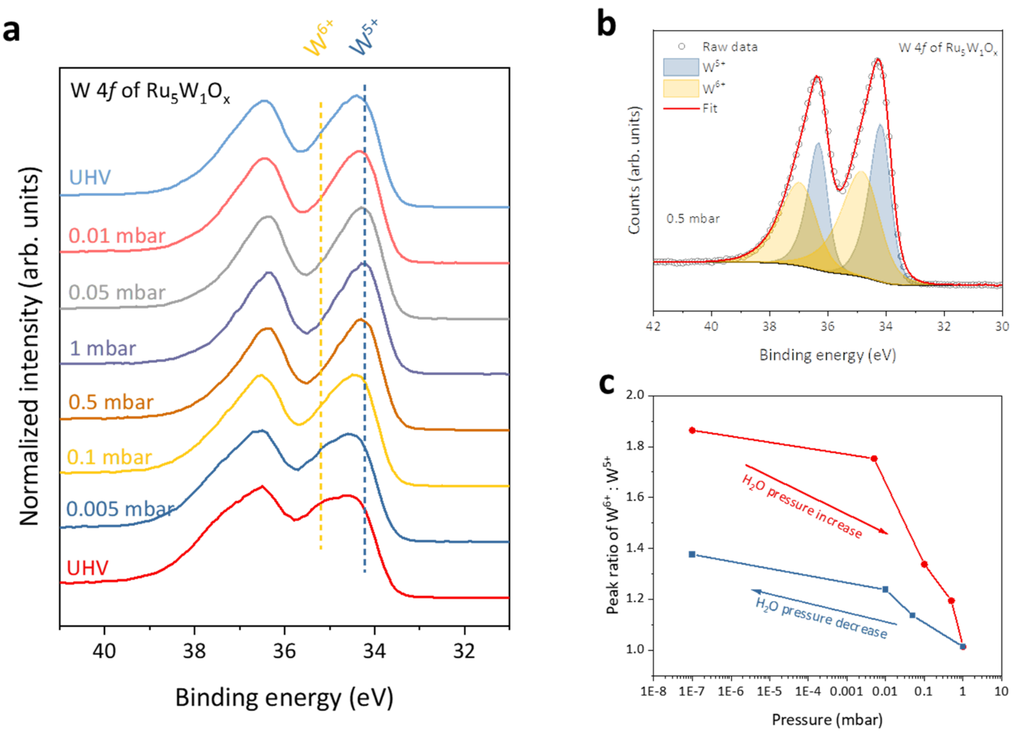

Supplementary Figure 31 | Analysis of W 4*f* XPS spectra of $Ru_5W_1O_x$. (a) The spectra at different water vapor pressures. **(b)** The typical deconvolution of W 4*f* XPS spectra of $Ru_5W_1O_x$. **(c)** The W^{6+}:W^{5+} ratio changes along with the water vapor pressure changes. At higher pressure, the content of W^{5+} increases, indicating the water molecules dissociatively adsorbed on the catalyst's surface by transferring protons to W-O_{bri}-Ru.

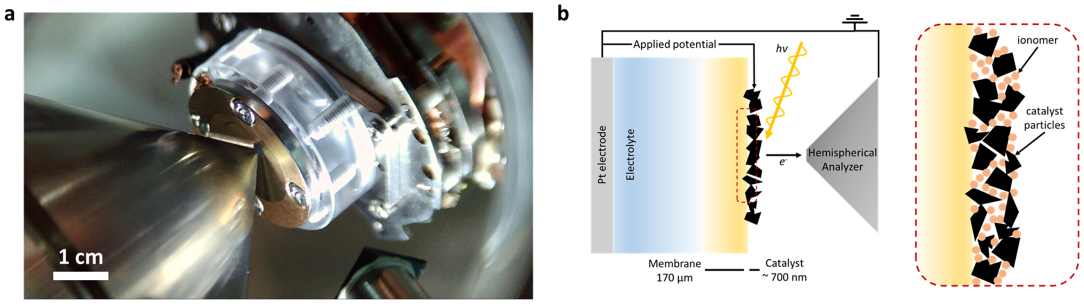

Supplementary Figure 32 | In situ electrochemical XPS setup. (a) The optical image of electrochemical cell in NAP-XPS chamber. **(b)** A schematic of the in situ electrochemical measurements. The zoom area illustrates the major components at the measured electrochemical interface.

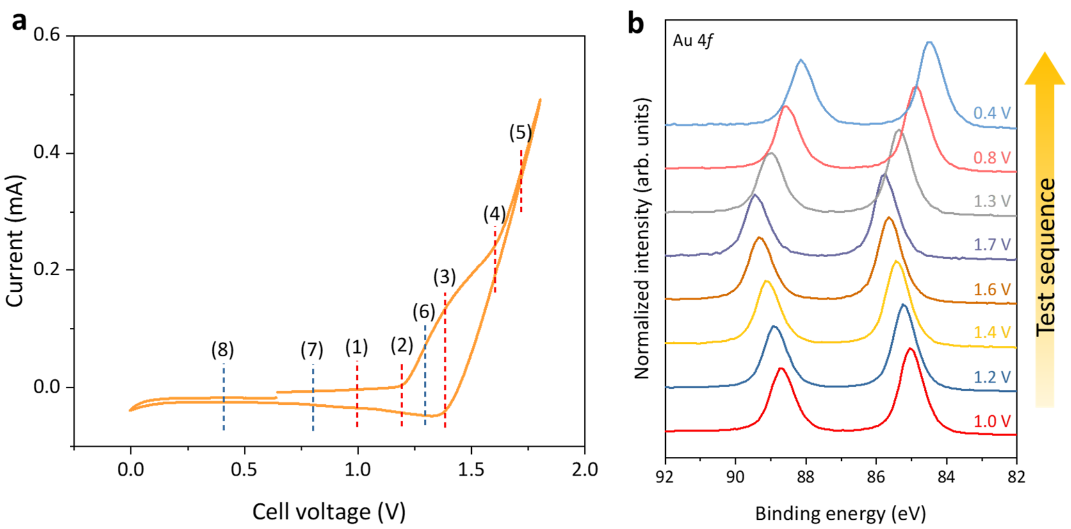

Supplementary Figure 33 | The electrochemistry of the in-situ XPS cell. (a) A typical CV curve of the electrochemical cell. The dash lines marked potentials for XPS spectra measurements. **(b)** The Au 4*f* XPS spectra under different applied potentials.

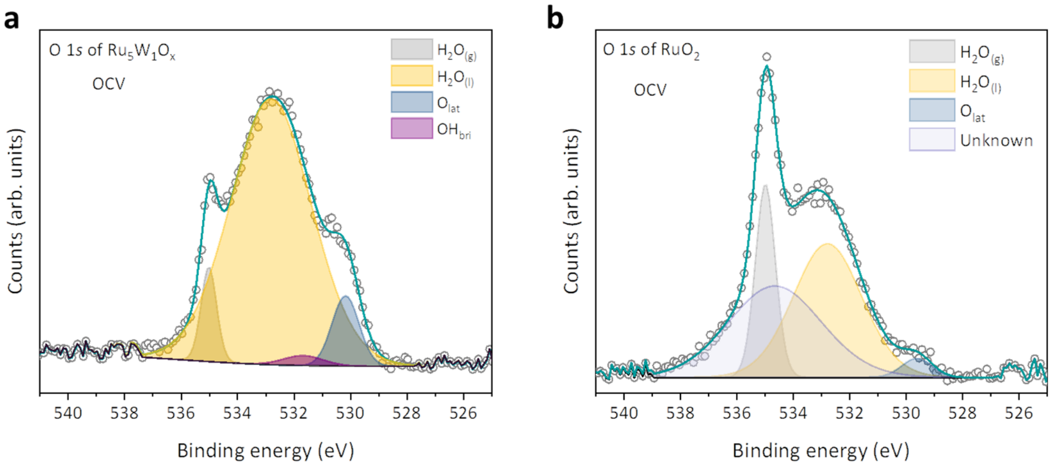

Supplementary Figure 34 | The O 1*s* XPS spectrum measured at open-circuit voltage. (a) $Ru_5W_1O_x$. **(b)** RuO_2. In RuO_2, the purple peak will diminish under X-ray exposure. We attribute this peak to organic contamination.

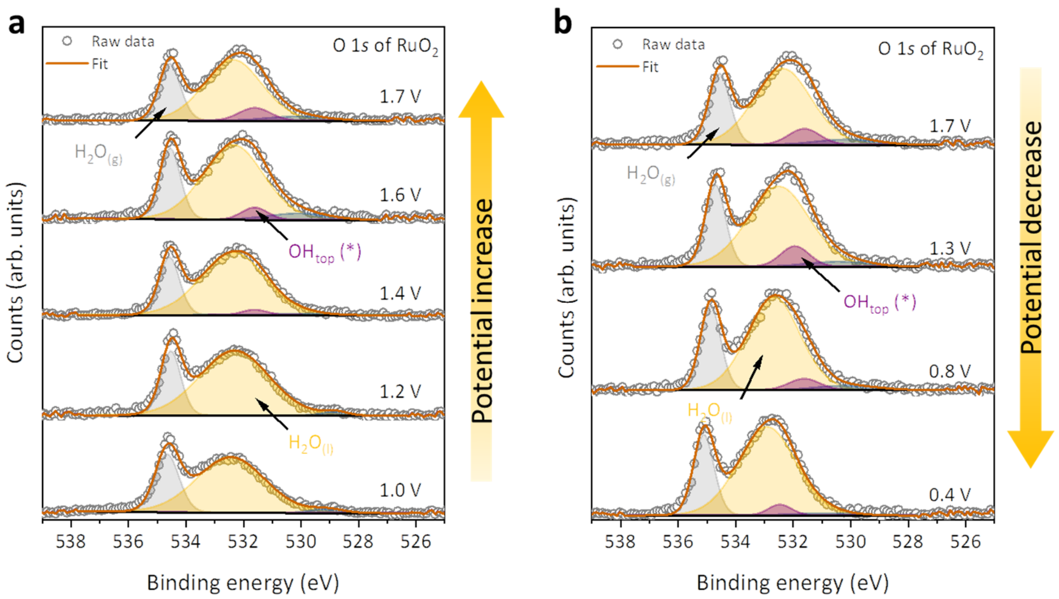

Supplementary Figure 35 | In situ electrochemical O 1*s* XPS spectra of RuO_2. (a) Spectra measured as the potential increase. **(b)** Spectra measured as the potential decrease. The binding energy of all spectra was calibrated according to the Au 4*f* peak at 84.0 eV. Since the surface of RuO_2 strongly interacted with water, it was difficult to identify the O_{lat} and OH_{bri} features from the in situ spectra. According to the deconvolution of O 1*s* spectra measured under the water vapor, we attributed the observed peak located at ~532 eV as the hydroxyls or water molecules adsorbed on the Ru_{CUS} sites. The pressure of the XPS chamber was maintained at 0.25 mbar by injecting water vapor.

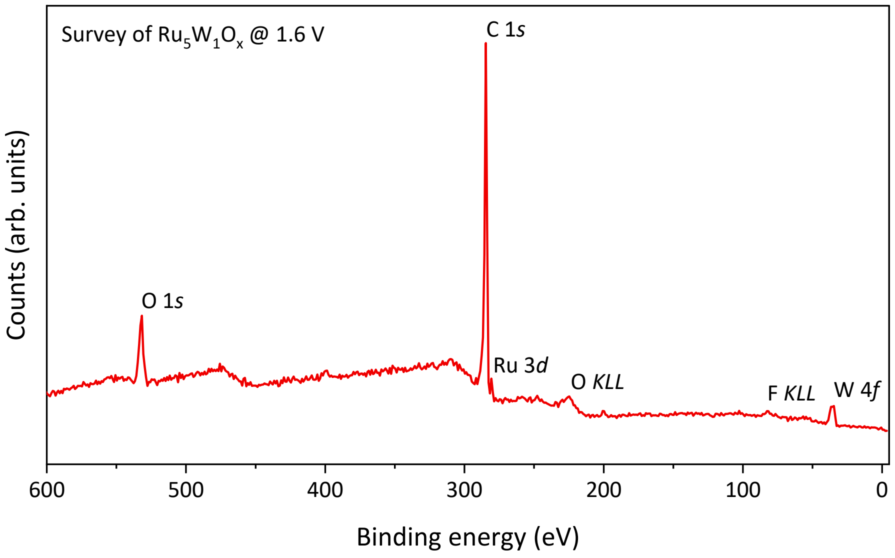

Supplementary Figure 36 | The XPS survey of $Ru_5W_1O_x$ at 1.6 V. The binding energy of the spectrum was calibrated according to the Au 4*f* peak at 84.0 eV. The pressure of the XPS chamber was maintained at 0.25 mbar by injecting water vapor.

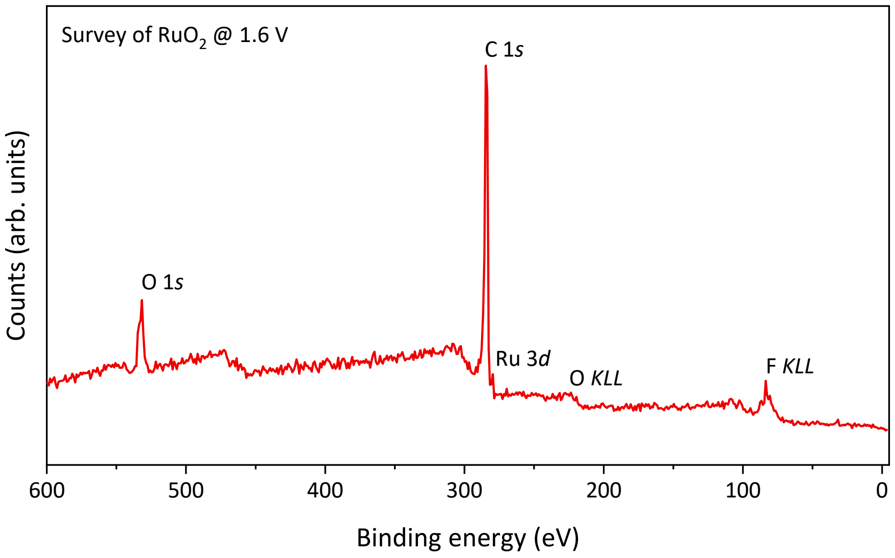

Supplementary Figure 37 | The XPS survey of RuO_2 at 1.6 V. The binding energy of the spectrum was calibrated according to the Au 4*f* peak at 84.0 eV. The pressure of the XPS chamber was maintained at 0.25 mbar by injecting water vapor.

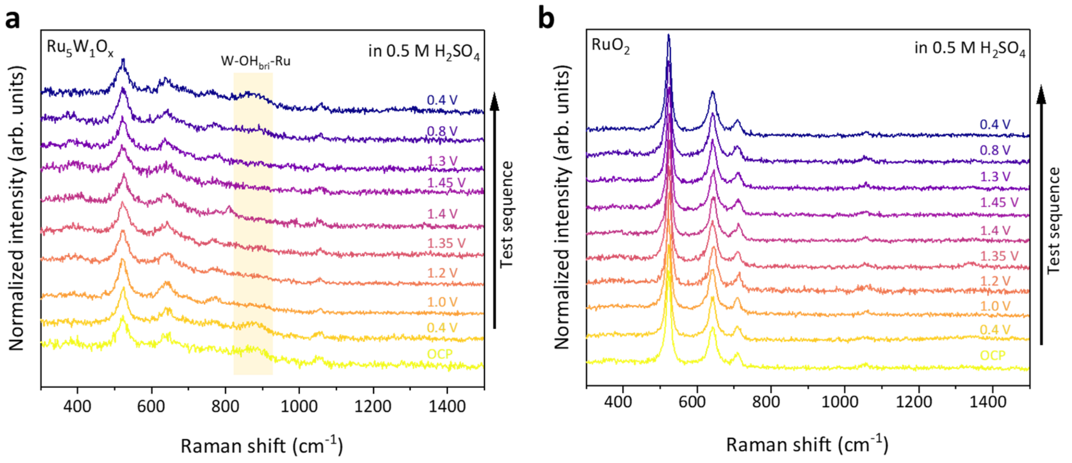

Supplementary Figure 38 | In situ Raman spectroscopy of (a) $Ru_5W_1O_x$ (b) RuO_2. In $Ru_5W_1O_x$, we observed the peak at *ca.* 880 cm^{-1} decreased along with the potential increase. The peak could recover when the potential decrease. The peak was not observed in powder samples, which indicated that this peak came from the interaction between water molecules and the catalyst surface. We, therefore, postulate that the peak change may come from the deprotonation of W-OH_{bri}-Ru.

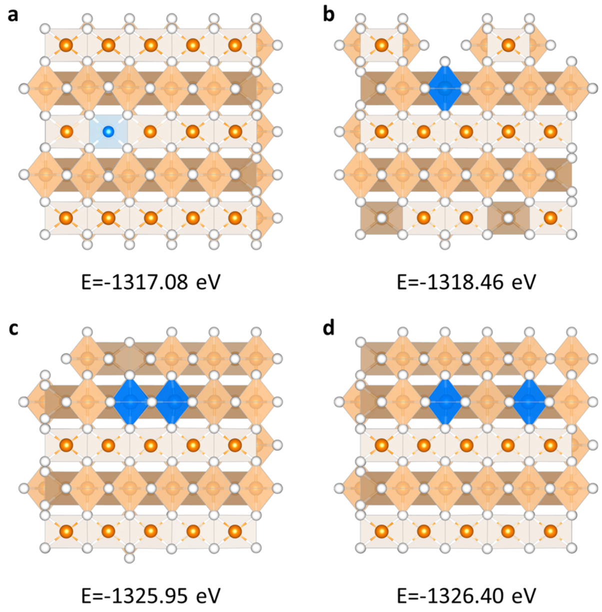

Supplementary Figure 39 | The DFT W-doping test on RuO_2 (110) surface. (a-b) Doping with a single W atom: The W atom prefers to substitute the bridge site **(b)** rather than the coordinatively unsaturated site **(a)**. **(c-d)** Doping with two W atoms: W atoms tend to locate discretely **(d)** instead of aggregately **(c)**. Orange balls – Ru, Blue balls – W, White balls – O. The orange and blue octahedrons represent RuO_6 and WO_6 octahedrons, respectively.

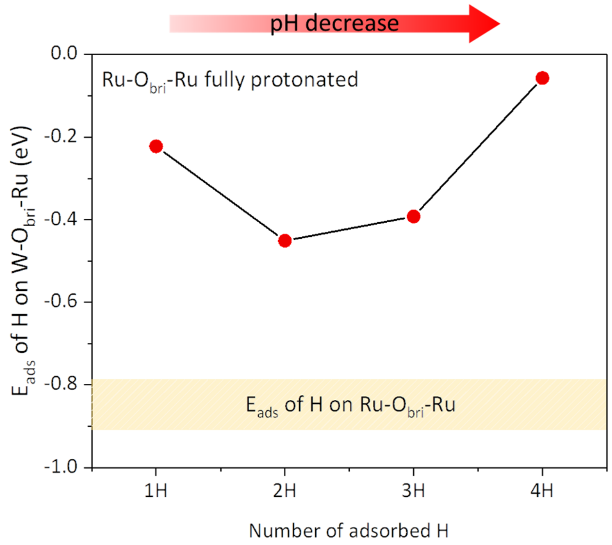

Supplementary Figure 40 | The H atom adsorption energy ($H^+ + e^-$) on the W-O$_{bri}$-Ru site with all Ru-O$_{bri}$-Ru being occupied by H. The E_{ads} of protons kept reducing along with the increase of hydrogen coverage and finally reached nearly thermal-neutral adsorption energy (-0.06 eV), indicating high proton mobility of W-doped RuO_2 in strong acidic electrolytes.

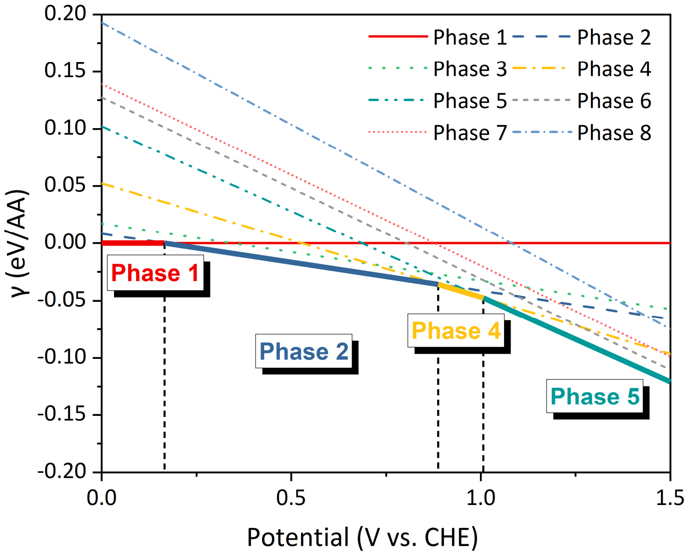

Supplementary Figure 41 | Surface phase diagram of W-doped RuO_2. The bold lines represented the most stable phases at different potentials. The surface phase diagram indicated that the O_{bri} on W-doped RuO_2 is fully deprotonated at ~0.8 V *vs.* computational hydrogen electrode (CHE), forming an O-terminated surface before the OER onset.

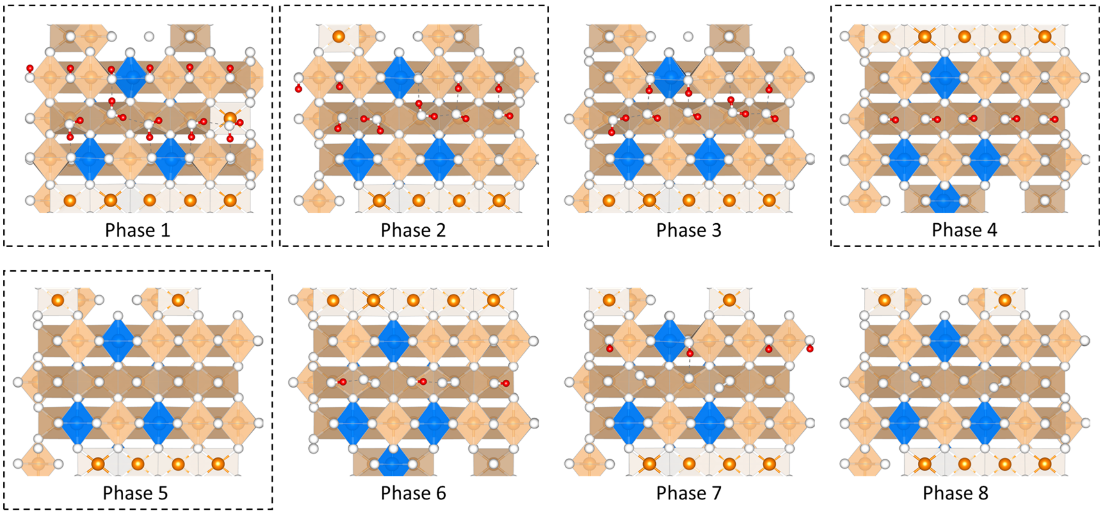

Supplementary Figure 42 | The surface phases considered in the phase diagram calculations. Phase 1, 2, 4, 5 were the most stable phases at different electrode potentials. Phase 1: The Ru_{CUS} sites were covered by water and the O_{bri} sites were fully protonated. Phase 2: The W-OH_{bri}-Ru sites were deprotonated, while the Ru-OH_{bri}-Ru sites were still H-covered. Phase 4: All bridging oxygen atoms were deprotonated. Phase 5: The adsorbed hydroxyls (OH_{top}) were deprotonated and formed an O-terminated surface.

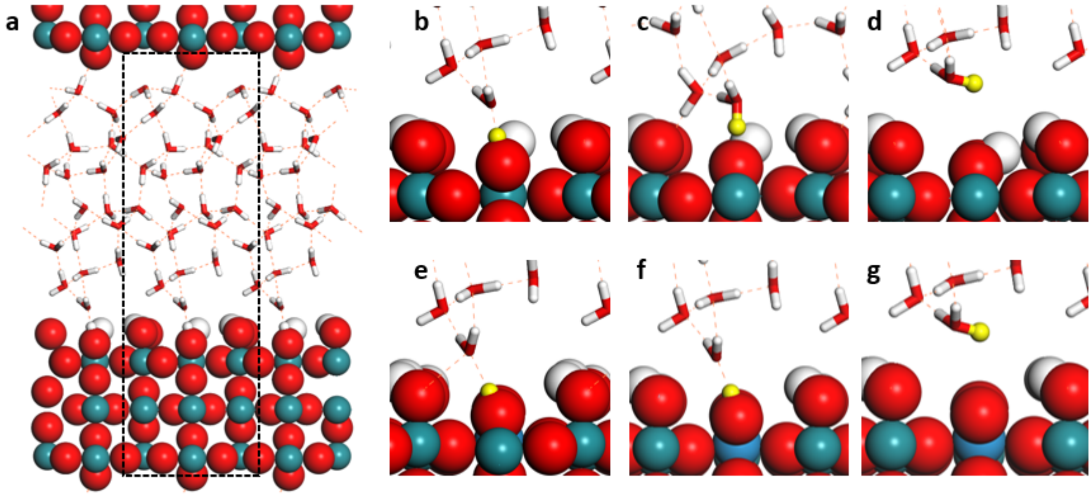

Supplementary Figure 43 | The explicit water models of water/(W-doped)RuO_2 interface. (a) Example of cell with explicit water used to compute surface phase diagram. The optimized configurations of the initial state (IS), the transition state (TS), and the final state (FS) of the proton transfer from the surface of **(b-d)** RuO_2 and **(e-g)** W-doped RuO_2. The transferred proton is highlighted and the bond length to the bridging oxygen atom is marked. The dashed yellow lines between water molecules indicate hydrogen bond interactions. Ru atoms are dark green, W as blue, O as red, and H as white.

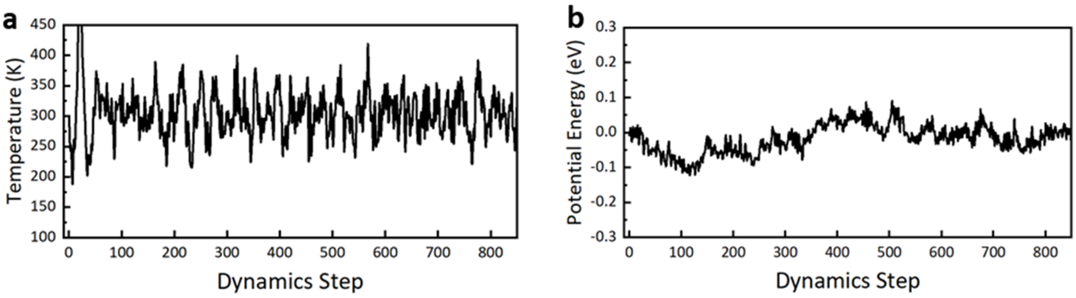

Supplementary Figure 44 | Details of AIMD simulations. (a) The temperature and **(b)** potential energy of 850 steps Ab initio molecular dynamics (AIMD) simulation.

Supplementary Tables

Supplementary Table 1 | Performance metrics of OER on different catalysts.

**Catalyst**	**η_{10}[1] (mV)**	**Mass specific activity (A g_{metal}^{-1})**	**Mass specific activity (A g_{Ru}^{-1})**	**TOF[2] ($metal^{-1}$ s^{-1})**
$Ru_3W_1O_x$	243±1	256±14	411±23	0.081±0.004
$Ru_5W_1O_x$	227±2	547±33	751±45	0.163±0.010
$Ru_7W_1O_x$	254±2	177±11	222±14	0.051±0.003
RuO_2	285±3	87±6	87±6	0.007±0.002
IrO_2	304±2	26±1	26±1	0.012±0.001

[1]η_{10}: Overpotential at 10 mA cm^{-2}

[2]Calculated according to the mass loading of all metal atoms at η = 300 mV.

Supplementary Table 2 | Summary of the surface area obtained by different methods.

Catalyst	S_{BET} (m^2 g^{-1})	[1]$S_{Hg\text{-}UPD}$ (m^2 g^{-1})	[2]S_{Cdl} (m^2 g^{-1})
$Ru_5W_1O_x$	53.86	33.78	76.1
RuO_2	9.85	8.03	33.4
IrO_2	11.98	6.48	24.2

We converted all the values to the same unit for a direct comparison.

[1]The surface area factor is 138.6 μC cm^{-2}.

[2]The surface area factor is 35 μF cm^{-2}.

Supplementary Table 3 | The deconvolution of O 1*s* peak of $Ru_5W_1O_x$ measured under different water vapor pressures. Temperature: 298 K.

Pressure (mbar)	Species	Position (eV)	FWHM	Area	Lineshape	OH_{bri}:O_{lat}
UHV	O_{lat}	529.90	1.43	143072	GL(10)	0.6271
	OH_{bri}	531.10	1.73	89718	GL(10)	
	OH_{top}	532.60	3.06	81791	GL(25)	
	$H_2O_{(g)}$	-	-	-	-	
0.005	O_{lat}	529.81	1.37	131740	GL(10)	0.7580
	OH_{bri}	531.01	1.82	99855	GL(10)	
	OH_{top}	532.85	2.46	80566	GL(25)	
	$H_2O_{(g)}$	-	-	-	-	
0.1	O_{lat}	529.67	1.30	117595	GL(10)	0.4636
	OH_{bri}	530.87	1.62	54513	GL(10)	
	OH_{top}	531.94	3.32	138497	GL(25)	
	$H_2O_{(g)}$	534.59	0.59	3709	GL(25)	
0.5	O_{lat}	529.48	1.21	67094	GL(10)	0.9826
	OH_{bri}	530.68	2.29	65925	GL(10)	
	OH_{top}	532.08	3.12	73168	GL(25)	
	$H_2O_{(g)}$	534.35	0.61	11526	GL(25)	
1	O_{lat}	529.44	1.16	41690	GL(10)	1.1909
	OH_{bri}	530.64	2.40	49674	GL(10)	
	OH_{top}	532.32	2.08	30220	GL(25)	
	$H_2O_{(g)}$	534.20	0.75	16402	GL(25)	
0.05*	O_{lat}	529.45	1.24	139704	GL(10)	1.1130
	OH_{bri}	530.65	2.42	155493	GL(10)	
	OH_{top}	532.61	2.75	68167	GL(25)	
	$H_2O_{(g)}$	-	-	-	-	
0.01*	O_{lat}	529.53	1.28	170651	GL(10)	0.3235
	OH_{bri}	530.73	1.56	55206	GL(10)	
	OH_{top}	531.62	3.47	153891	GL(25)	
	$H_2O_{(g)}$	-	-	-	-	
UHV*	O_{lat}	529.63	1.30	174631	GL(10)	0.4313
	OH_{bri}	530.83	1.64	75325	GL(10)	
	OH_{top}	531.80	3.45	126001	GL(25)	
	$H_2O_{(g)}$	-	-	-	-	

*Back to the low pressure.

Supplementary Table 4 | The deconvolution of O 1*s* peak of RuO_2 measured under different water vapor pressures. Temperature: 298 K.

Pressure (mbar)	Species	Position (eV)	FWHM	Area	Lineshape	OH_{bri}:O_{lat}
UHV	O_{lat}	529.28	0.89	43517	GL(10)	3.0280
	OH_{bri}	530.78	2.52	131771	GL(10)	
	OH_{top}	533.57	2.34	18659	GL(25)	
	$H_2O_{(g)}$				-	
0.005	O_{lat}	529.30	0.78	30245	GL(10)	4.3277
	OH_{bri}	530.80	2.50	130892	GL(10)	
	OH_{top}	533.15	2.65	28559	GL(25)	
	$H_2O_{(g)}$				-	
0.1	O_{lat}	529.31	0.77	24467	GL(10)	4.2657
	OH_{bri}	530.81	2.39	104371	GL(10)	
	OH_{top}	532.86	2.50	35572	GL(25)	
	$H_2O_{(g)}$	535.02	0.90	3745	GL(25)	
0.5	O_{lat}	529.30	0.73	14089	GL(10)	4.5400
	OH_{bri}	530.80	2.47	63967	GL(10)	
	OH_{top}	532.81	2.56	28048	GL(25)	
	$H_2O_{(g)}$	535.02	0.63	7216	GL(25)	
1	O_{lat}	529.30	0.73	7762	GL(10)	4.5228
	OH_{bri}	530.80	2.43	31802	GL(10)	
	OH_{top}	532.78	2.32	15224	GL(25)	
	$H_2O_{(g)}$	535.06	0.61	7762	GL(25)	
UHV*	O_{lat}	529.31	0.75	23042	GL(10)	4.5537
	OH_{bri}	530.81	2.52	104929	GL(10)	
	OH_{top}	532.69	3.39	39295	GL(25)	
	$H_2O_{(g)}$	-	-	-	-	

*Back to the low pressure.

Supplementary Table 5 | The deconvolution of W 4*f* peak of $Ru_5W_1O_x$ measured under different water vapor pressures. Temperature: 298 K.

Pressure (mbar)	Species	Position (eV)	FWHM	Area	Lineshape	W^{6+}:W^{5+}
UHV	W^{5+}	34.24	0.89	45349	LF(0.4,1,20,280)	1.86406
	W^{5+}, sat	36.39	0.80	34012	LF(0.4,1,20,280)	
	W^{6+}	35.08	1.54	84534	LF(0.4,1,20,280)	
	W^{6+}, sat	37.23	1.45	63400	LF(0.4,1,20,280)	
0.005	W^{5+}	34.23	0.87	45549	LF(0.4,1,20,280)	1.75311
	W^{5+}, sat	36.38	0.77	34161	LF(0.4,1,20,280)	
	W^{6+}	35.00	1.52	79852	LF(0.4,1,20,280)	
	W^{6+}, sat	37.15	1.42	59889	LF(0.4,1,20,280)	
0.1	W^{5+}	34.20	0.85	51788	LF(0.4,1,20,280)	1.33777
	W^{5+}, sat	36.35	0.78	38841	LF(0.4,1,20,280)	
	W^{6+}	34.91	1.50	69281	LF(0.4,1,20,280)	
	W^{6+}, sat	37.06	1.41	51960	LF(0.4,1,20,280)	
0.5	W^{5+}	34.09	0.80	42337	LF(0.4,1,20,280)	1.19458
	W^{5+}, sat	36.24	0.74	31752	LF(0.4,1,20,280)	
	W^{6+}	34.68	1.43	50575	LF(0.4,1,20,280)	
	W^{6+}, sat	36.83	1.36	37931	LF(0.4,1,20,280)	
1	W^{5+}	34.07	0.81	39405	LF(0.4,1,20,280)	1.0136
	W^{5+}, sat	36.22	0.75	29553	LF(0.4,1,20,280)	
	W^{6+}	34.63	1.37	39941	LF(0.4,1,20,280)	
	W^{6+}, sat	36.78	1.29	29955	LF(0.4,1,20,280)	
0.05*	W^{5+}	34.09	0.80	61995	LF(0.4,1,20,280)	1.13621
	W^{5+}, sat	36.24	0.74	46496	LF(0.4,1,20,280)	
	W^{6+}	34.65	1.42	70439	LF(0.4,1,20,280)	
	W^{6+}, sat	36.80	1.33	52829	LF(0.4,1,20,280)	
0.01*	W^{5+}	34.13	0.83	61670	LF(0.4,1,20,280)	1.23781
	W^{5+}, sat	36.28	0.75	46252	LF(0.4,1,20,280)	
	W^{6+}	34.75	1.45	76336	LF(0.4,1,20,280)	
	W^{6+}, sat	36.90	1.32	57252	LF(0.4,1,20,280)	
UHV*	W^{5+}	34.14	0.84	59607	LF(0.4,1,20,280)	1.37635
	W^{5+}, sat	36.29	0.76	44705	LF(0.4,1,20,280)	
	W^{6+}	34.79	1.43	82040	LF(0.4,1,20,280)	
	W^{6+}, sat	36.94	1.32	61530	LF(0.4,1,20,280)	

*Back to the low pressure.

**Fitting criteria: The BE of the $4f^{5/2}$ peak was restricted to +2.15 eV of the main peak ($4f^{7/2}$). The peak area ratio of $4f^{7/2}$: $4f^{5/2}$ is set to 4:3 according to the spin-orbit splitting of *f* electrons.

Supplementary Table 6 | The deconvolution of O 1*s* peak of $Ru_5W_1O_x$ measured under different potentials. Pressure: 0.25 mbar. Temperature: 298 K.

Potential (V)	Species	Position (eV)	FWHM	Area	Lineshape
OCV	O_{lat}	530.17	1.13	5387	GL(10)
	OH_{bri}	531.67	1.69	1143	GL(10)
	$H_2O_{(l)}$	532.81	3.39	61678	GL(25)
	$H_2O_{(g)}$	535.01	0.65	4234	GL(25)
1.0	O_{lat}	529.82	1.33	6158	GL(10)
	OH_{bri}	531.32	2.02	8383	GL(10)
	$H_2O_{(l)}$	532.57	2.36	53892	GL(25)
	$H_2O_{(g)}$	534.77	0.88	6847	GL(25)
1.2	O_{lat}	529.96	1.36	5342	GL(10)
	OH_{bri}	531.46	1.66	4716	GL(10)
	$H_2O_{(l)}$	532.55	2.32	50389	GL(25)
	$H_2O_{(g)}$	534.75	0.96	6277	GL(25)
1.4	O_{lat}	530.06	1.50	5471	GL(10)
	OH_{bri}	531.56	1.30	2149	GL(10)
	$H_2O_{(l)}$	532.50	2.28	50961	GL(25)
	$H_2O_{(g)}$	534.70	1.07	7289	GL(25)
1.6	O_{lat}	529.99	1.48	4397	GL(10)
	OH_{bri}	531.49	2.23	126	GL(10)
	$H_2O_{(l)}$	532.45	2.31	52290	GL(25)
	$H_2O_{(g)}$	534.65	0.99	6445	GL(25)
1.7	O_{lat}	529.96	1.66	4074	GL(10)
	OH_{bri}	531.46	1.50**	0**	GL(10)
	$H_2O_{(l)}$	532.46	2.28	50768	GL(25)
	$H_2O_{(g)}$	534.66	1.10	7251	GL(25)
1.3*	O_{lat}	529.91	1.25	2592	GL(10)
	OH_{bri}	531.41	1.86	576	GL(10)
	$H_2O_{(l)}$	532.49	2.35	51358	GL(25)
	$H_2O_{(g)}$	534.69	1.00	6275	GL(25)
0.8*	O_{lat}	529.97	1.43	2499	GL(10)
	OH_{bri}	531.47	1.99	1701	GL(10)
	$H_2O_{(l)}$	532.55	2.26	48413	GL(25)
	$H_2O_{(g)}$	534.75	0.96	6446	GL(25)
0.4*	O_{lat}	530.10	1.16	2447	GL(10)
	OH_{bri}	531.60	1.39	2109	GL(10)
	$H_2O_{(l)}$	532.59	2.17	45649	GL(25)
	$H_2O_{(g)}$	534.79	1.01	6864	GL(25)

*Back to the low potential.

**Due to the decrease of OH_{bri} species, no peak could be identified from the deconvolution of spectra.

Supplementary Table 7 | The deconvolution of O 1*s* peak of RuO_2 measured under different potentials. Pressure: 0.25 mbar. Temperature: 298 K.

Potential (V)	Species	Position (eV)	FWHM	Area	Lineshape
OCV	O_{lat}	529.62	1.26	1117	GL(10)
	OH_{bri}***	534.64	3.89	16885	GL(25)
	$H_2O_{(l)}$	532.78	2.88	18281	GL(25)
	$H_2O_{(g)}$	534.98	0.84	7778	GL(25)
1.0	O_{lat}	529.06	1.32	953	GL(10)
	OH_{bri}	530.56	1.09**	0**	GL(10)
	$H_2O_{(l)}$	532.44	2.79	29027	GL(25)
	$H_2O_{(g)}$	534.64	0.99	10656	GL(25)
1.2	O_{lat}	528.80	0.94	574	GL(10)
	OH_{bri}	530.30	0.51**	0**	GL(10)
	$H_2O_{(l)}$	532.31	2.84	29306	GL(25)
	$H_2O_{(g)}$	534.51	0.82	8437	GL(25)
1.4	O_{lat}	530.10	2.50	495	GL(10)
	OH_{bri}	531.60	1.01	837	GL(10)
	$H_2O_{(l)}$	532.36	2.65	26652	GL(25)
	$H_2O_{(g)}$	534.56	0.84	7855	GL(25)
1.6	O_{lat}	530.10	2.50	2078	GL(10)
	OH_{bri}	531.60	1.07	1707	GL(10)
	$H_2O_{(l)}$	532.34	2.49	24533	GL(25)
	$H_2O_{(g)}$	534.54	0.85	8013	GL(25)
1.7	O_{lat}	530.10	2.50	1639	GL(10)
	OH_{bri}	531.60	1.27	2553	GL(10)
	$H_2O_{(l)}$	532.35	2.37	22924	GL(25)
	$H_2O_{(g)}$	534.55	0.89	8074	GL(25)
1.3*	O_{lat}	530.42	2.50	1324	GL(10)
	OH_{bri}	531.92	1.17	2585	GL(10)
	$H_2O_{(l)}$	532.49	2.52	22145	GL(25)
	$H_2O_{(g)}$	534.69	0.83	7489	GL(25)
0.8*	O_{lat}	530.10	2.50	1210	GL(10)
	OH_{bri}	532.60	1.27	1548	GL(10)
	$H_2O_{(l)}$	532.67	2.15	22080	GL(25)
	$H_2O_{(g)}$	534.87	0.85	7949	GL(25)
0.4*	O_{lat}	530.95	2.50	571	GL(10)
	OH_{bri}	532.45	0.95	1108	GL(10)
	$H_2O_{(l)}$	532.90	2.36	22902	GL(25)
	$H_2O_{(g)}$	535.10	0.85	7567	GL(25)

*Back to the low potential.

**No peak could be identified from the deconvolution of spectra

***Unknown species. Which may come from contamination.

Supplementary Table 8 | Performance of iridium-free OER catalysts in acidic electrolytes.

Catalyst	η_{10}* (mV)	η_{10} after long-term operation (mV)	Degradation rate (mV h^{-1})	Reference
$Ru_5W_1O_x$	227	235 (after 550 h)	0.014	This work
IrO_2	304	313 (after 250 h)	0.036	This work
$12Ru/MnO_2$	161	324 (after 200 h)	0.82	Ref.[24]
$Ru_1\text{-}Pt_3Cu$	220	259 (after 28 h)	1.39	Ref.[25]
$Mn\text{-}RuO_2$	158	359 (after 10 h)	20.1	Ref.[26]
$Cr_{0.6}Ru_{0.4}O_2$	178	249 (after 10 h)	7.1	Ref.[27]
$Cu\text{-}RuO_2$	188	271 (after 8 h)	10.4	Ref.[28]
$RuO_2/(CoMn)_3O_4$	270	401 (after 24 h)	5.46	Ref.[29]
RuO_2 NSs	199	231 (after 6 h)	5.33	Ref.[30]
$W_{0.2}Er_{0.1}Ru_{0.7}O_{2-\delta}$	168	260 (after 500 h)	0.184	Ref.[31]

*η_{10}: The overpotential at 10 mA cm^{-2}.

Supplementary References

1 Sun, S., Li, H. and Xu, Z. J. Impact of surface area in evaluation of catalyst activity. *Joule* **2**, 1024-1027 (2018).

2 McCrory, C. C., Jung, S., Peters, J. C. and Jaramillo, T. F. Benchmarking heterogeneous electrocatalysts for the oxygen evolution reaction. *J. Am. Chem. Soc.* **135**, 16977-16987 (2013).

3 Stevens, M. B. *et al.* Measurement techniques for the study of thin film heterogeneous water oxidation electrocatalysts. *Chem. Mater.* **29**, 120-140 (2016).

4 Bai, L., Hsu, C.-S., Alexander, D. T. L., Chen, H. M. and Hu, X. Double-atom catalysts as a molecular platform for heterogeneous oxygen evolution electrocatalysis. *Nat. Energy* **6**, 1054-1066 (2021).

5 Over, H. Fundamental studies of planar single-crystalline oxide model electrodes (RuO_2, IrO_2) for acidic water splitting. *ACS Catal.* **11**, 8848-8871 (2021).

6 Zhang, J. *et al.* Advances in thermodynamic-kinetic model for analyzing the oxygen evolution reaction. *ACS Catal.* **10**, 8597-8610 (2020).

7 Exner, K. S., Sohrabnejad-Eskan, I., Anton, J., Jacob, T. and Over, H. Full free energy diagram of an electrocatalytic reaction over a single-crystalline model electrode. *ChemElectroChem* **4**, 2902-2908 (2017).

8 Exner, K. S., Sohrabnejad-Eskan, I. and Over, H. A universal approach to determine the free energy diagram of an electrocatalytic reaction. *ACS Catal.* **8**, 1864-1879 (2018).

9 Fang, Y. H. and Liu, Z. P. Mechanism and Tafel lines of electro-oxidation of water to oxygen on RuO_2(110). *J. Am. Chem. Soc.* **132**, 18214-18222 (2010).

10 Rao, R. R. *et al.* Operando identification of site-dependent water oxidation activity on ruthenium dioxide single-crystal surfaces. *Nat. Catal.* **3**, 516-525 (2020).

11 Rao, R. R. *et al.* Surface orientation dependent water dissociation on rutile ruthenium dioxide. *J. Phys. Chem. C* **122**, 17802-17811 (2018).

12 Lyons, M. E. and Floquet, S. Mechanism of oxygen reactions at porous oxide electrodes. Part 2--Oxygen evolution at RuO_2, IrO_2 and $Ir_xRu_{1-x}O_2$ electrodes in aqueous acid and alkaline solution. *Phys. Chem. Chem. Phys.* **13**, 5314-5335 (2011).

13 Falling, L. J. *et al.* Graphene-capped liquid thin films for electrochemical operando X-ray spectroscopy and scanning electron microscopy. *ACS Appl. Mater. Interfaces* **12**, 37680-37692 (2020).

14 Favaro, M. *et al.* Unravelling the electrochemical double layer by direct probing of the solid/liquid interface. *Nat. Commun.* **7**, 12695 (2016).

15 Shah, D. *et al.* Liquid water, by near-ambient pressure XPS. *Surf. Sci. Spectra* **26** (2019).

16 Truhlar, D. G., Garrett, B. C. and Klippenstein, S. J. Current status of transition-state theory. *J. Phys. Chem.* **100**, 12771-12800 (1996).

17 Lamoureux, P. S., Singh, A. R. and Chan, K. pH effects on hydrogen evolution and oxidation over Pt(111): Insights from first-principles. *ACS Catal.* **9**, 6194-6201 (2019).

18 Tian, Y. *et al.* Visualizing Eigen/Zundel cations and their interconversion in monolayer water on metal surfaces. *Science* **377**, 315-319 (2022).

19 Huang, Y. S. and Pollak, F. H. Raman investigation of rutile RuO_2. *Solid State Commun.* **43**, 921-924 (1982).

20 Li, W.-Q. *et al.* Identification of the molecular pathways of RuO_2 electroreduction by in-situ electrochemical surface-enhanced Raman spectroscopy. *J. Catal.* **400**, 367-371 (2021).

21 Daniel, M. F., Desbat, B., Lassegues, J. C., Gerand, B. and Figlarz, M. Infrared and Raman study of WO_3 tungsten trioxides and WO_3, xH_2O tungsten trioxide tydrates. *J. Solid State Chem.* **67**, 235-247 (1987).

22 Beck, F., Junge, H. and Krohn, H. Graphite intercalation compounds as positive electrodes in galvanic cells. *Electrochim. Acta* **26**, 799-809 (1981).

23 Sullivan, M. G. *et al.* Electrochemically modified glassy carbon for capacitor electrodes characterization of thick anodic layers by cyclic voltammetry, differential electrochemical mass spectrometry, spectroscopic ellipsometry, X-ray photoelectron spectroscopy, FTIR, and AFM. *J. Electrochem. Soc.* **147**, 2636-2643 (2000).

24 Lin, C. *et al.* In-situ reconstructed Ru atom array on α-MnO_2 with enhanced performance for acidic water oxidation. *Nat. Catal.* **4**, 1012-1023 (2021).

25 Yao, Y. *et al.* Engineering the electronic structure of single atom Ru sites via compressive strain boosts acidic water oxidation electrocatalysis. *Nat. Catal.* **2**, 304-313 (2019).

26 Chen, S. *et al.* Mn-doped RuO_2 nanocrystals as highly active electrocatalysts for enhanced oxygen evolution in acidic media. *ACS Catal.* **10**, 1152-1160 (2019).

27 Lin, Y. *et al.* Chromium-ruthenium oxide solid solution electrocatalyst for highly efficient oxygen evolution reaction in acidic media. *Nat. Commun.* **10**, 162 (2019).

28 Su, J. *et al.* Assembling ultrasmall copper-doped ruthenium oxide nanocrystals into hollow porous polyhedra: Highly robust electrocatalysts for oxygen evolution in acidic media. *Adv. Mater.*, e1801351 (2018).

29 Niu, S. *et al.* Low Ru loading $RuO_2/(Co,Mn)_3O_4$ nanocomposite with modulated electronic structure for efficient oxygen evolution reaction in acid. *Appl. Catal. B-Environ.* **297**, 120442 (2021).

30 Zhao, Z. L. *et al.* Boosting the oxygen evolution reaction using defect-rich ultra-thin ruthenium oxide nanosheets in acidic media. *Energy Environ. Sci.* **13**, 5143-5151 (2020).

31 Hao, S. *et al.* Dopants fixation of ruthenium for boosting acidic oxygen evolution stability and activity. *Nat. Commun.* **11**, 5368 (2020).
